# Crystal structures of (*S*)-3-{1-[(4-chloro­phen­yl)sulfon­yl]piperidin-2-yl}pyridine, (*S*)-3-[1-(4-methyl­phen­yl)piperidin-2-yl]pyridine, (*S*)-3-{1-[(4-meth­oxy­phen­yl)sulfon­yl]piperidin-2-yl}pyridine and (*S*)-3-{1-[(3,4-di­methyl­phen­yl)sulfon­yl]piperidin-2-yl}pyridine

**DOI:** 10.1107/S2056989026004524

**Published:** 2026-05-07

**Authors:** R. Ya. Okmanov, M. I. Olimova, I. E. Yuldashova, G. Z. Azimova, F. A. Pulatova

**Affiliations:** a S. Yunusov Institute of the Chemistry of Plant Substances Academy of Sciences of the Republic of Uzbekistan Mirzo Ulugbek str 77 Tashkent 100170 Uzbekistan; b National University of Uzbekistan named after Mirzo Ulugbek Universitet str 4 Almazar district Tashkent 100174 Uzbekistan; c Tashkent Pharmaceutical institute, Mirabad district, Aybek str., 45, Tashkent, 100015, Uzbekistan; Indian Institute of Science Education and Research Bhopal, India

**Keywords:** alkaloid anabazin, 3-(piperidin-2-yl)pyridine, aryl­sulfon­yl, crystal structure

## Abstract

In the presence of tri­methyl­amine, new compounds were obtained by aryl­sulfonyl­ation of 3-(piperidin-2-yl)pyridine (the alkaloid anabasine), namely: (*S*)-3-{1-[(4-chloro­phen­yl)sulfon­yl]piperidin-2-yl}pyridine, C_16_H_17_ClN_2_O_2_S (**1**); (*S*)-3-[1-(4-methyl­phen­yl)piperidin-2-yl]pyridine, C_17_H_20_N_2_O_2_S (**2**); (*S*)-3-{1-[(4-meth­oxy­phen­yl)sulfon­yl]piperidin-2-yl}pyridine, C_17_H_20_N_2_O_3_S (**3**); and (*S*)-3-{1-[(3,4-di­methyl­phen­yl)sulfon­yl]piperidin-2-yl}pyridine, C_18_H_22_N_2_O_2_S (**4**). In the crystal structures, the spatial arrangement of the pyridine and piperidine rings around the C*sp*^2^—C* bond, as well as the orientation of the benzene ring of the aryl­sulfonyl group relative to the anabasine moiety, are analyzed.

## Abstract 2

The crystal structures of (*S*)-3-{1-[(4-chloro­phen­yl)sulfon­yl]piperidin-2-yl}pyridine, (*S*)-3-[1-(4-methyl­phen­yl)piperidin-2-yl]pyridine, (*S*)-3-{1-[(4-meth­oxy­phen­yl)sulfon­yl]piperidin-2-yl}pyridine and (*S*)-3-{1-[(3,4-di­methyl­phen­yl)sulfon­yl]piperidin-2-yl}pyridine are compared. The similarities in the spatial structures of the mol­ecules are explained, particularly the mutual arrangement of the pyridine and piperidine rings, as well as the positioning of the aryl­sulfonyl group relative to the piperidine ring.

## 1. Chemical context

Alkaloids are natural organic compounds containing nitro­gen atoms that are synthesized by plants. They play an important role in pharmacology, toxicology, and medicinal chemistry (Dewick, 2009 ▸; Daly, 2005 ▸).

Anabasine (C_10_H_14_N_2_) is a natural alkaloid with a pyridine–piperidine structure, primarily found in *Anabasis aphylla* L. (Ujváry, 2010 ▸). Pharmacological studies have shown that anabasine exhibits a stimulating effect at low doses, whereas at high doses it produces strong toxic effects. High concentrations may lead to paralysis of the nervous system, depression of the respiratory center, and death (Kuete, 2014 ▸). For this reason, anabasine is classified as a toxic alkaloid and its use is restricted. Historically, anabasine was used as an insecticide in agriculture; however, due to its high toxicity, it is not widely applied in practice today (Amtaghri *et al.*, 2025 ▸).

In addition to being isolated from plants, synthetic methods for obtaining anabasine have also been developed (Felpin *et al.*, 2000 ▸). Numerous reactions have been carried out based on anabasine, most of which are focused on the synthesis of N-derivatives (Kulakov, 2010 ▸; Slyn’ko *et al.*, 2013 ▸; Bakbardina *et al.*, 2006 ▸). Although the main objective of these studies has been the synthesis of new biologically active compounds, particular emphasis has been placed on obtaining less toxic and biologically selective derivatives (Mukusheva *et al.*, 2022 ▸; Artyushin *et al.*, 2016 ▸).

Aryl­sulfonyl­ation reactions are typically carried out at room temperature in various solvents in the presence of tri­ethyl­amine or sodium hydroxide. According to the literature, the reaction time varies, mainly depending on the reactivity of the reagents involved. Structural studies of the synthesized aryl­sulfonyl products using X-ray crystallographic analysis have provided inter­esting and distinctive results (Abdireymov *et al.*, 2011 ▸; Okmanov *et al.*, 2022 ▸, 2023 ▸).
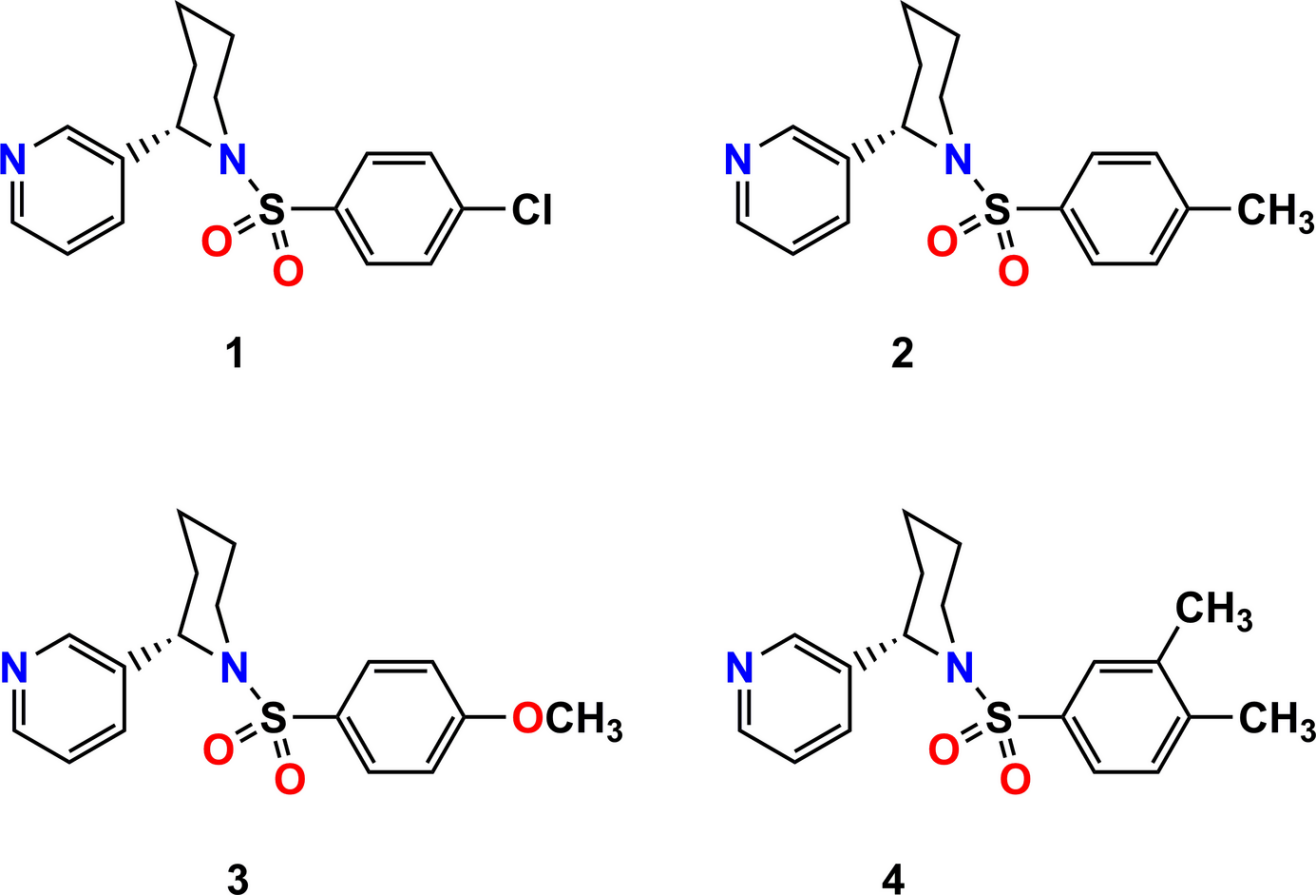


## 2. Structural commentary

**Figure 1:**
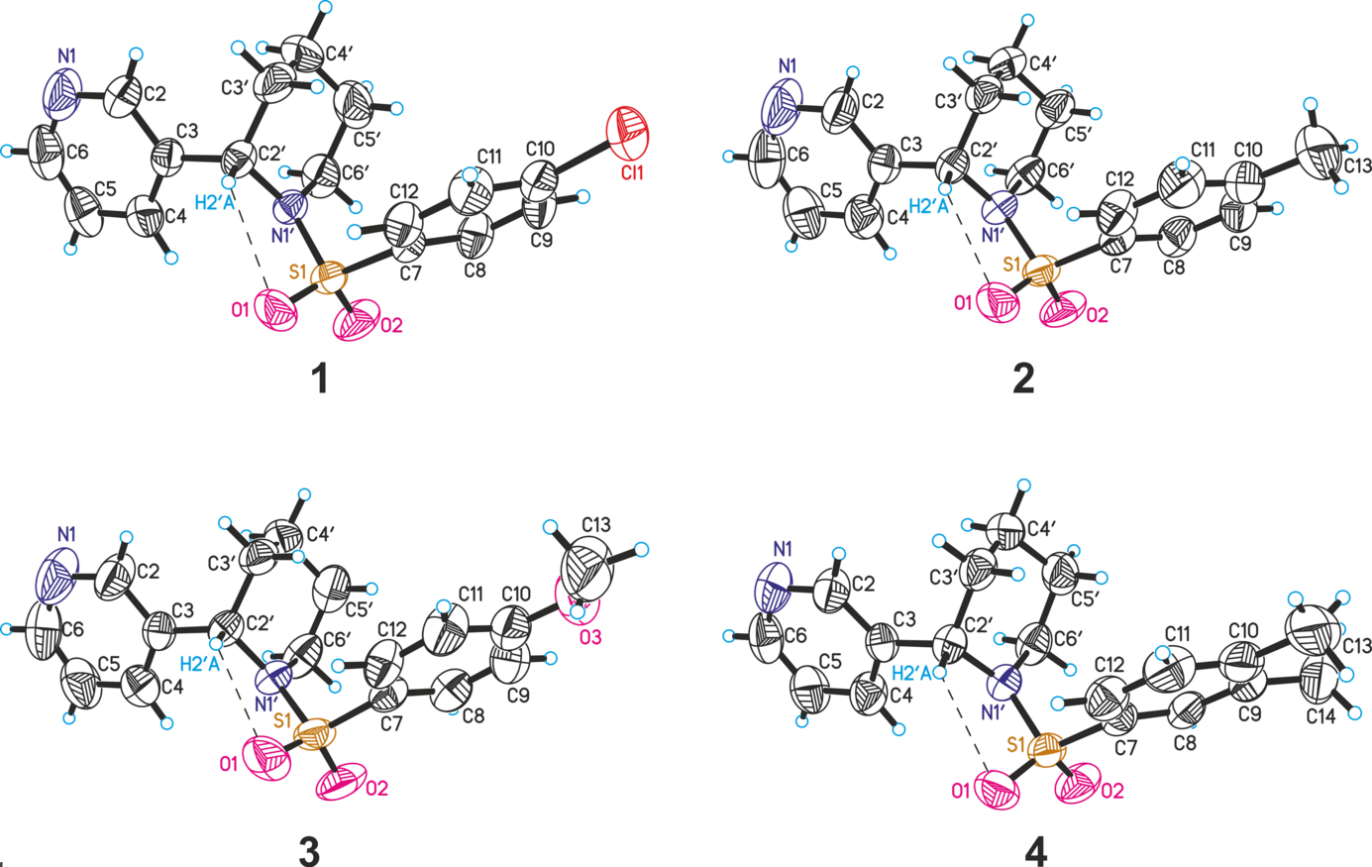
The mol­ecular structures of the title compounds drawn at 50% probability ellipsoids.

The asymmetric unit of all the structures consists of a single mol­ecule (Fig. 1 ▸). In the anabasine fragment, the piperidine rings adopt a chair conformation, and the spatial orientation of the pyridine ring relative to it is observed to be axial. In structures **1**–**4**, the relative arrangement of the pyridine and piperidine rings around the C*sp*^2^—C* bond differs slightly. This can be explained by the values of the N1′—C*—C—C torsion angles. The torsion angle values are 34.0 (4)° for **1**, 31.3 (4)° for **2**, 25.2 (5)° for **3**, and 28.4 (5)° for **4**. According to literature sources, in anabasine derivatives with various substituents, these torsion angles range from 17 to 82° (Kulakov *et al.*, 2010 ▸; Wojciechowska-Nowak *et al.*, 2007 ▸).

When comparing the N(pyridine)⋯N(piperidine) distances in the anabasine fragment, it is observed that they are very similar: 4.799 (4) Å for **1**, 4.819 (4) Å for **2**, 4.833 (5) Å for **3**, and 4.832 (4) Å for **4**. In other anabasine derivatives, depending on the spatial arrangement of the piperidine and pyridine rings, the N⋯N distances have been reported to range from 4.29 to 4.86 Å (Wojciechowska-Nowak *et al.*, 2007 ▸).

**Figure 2:**
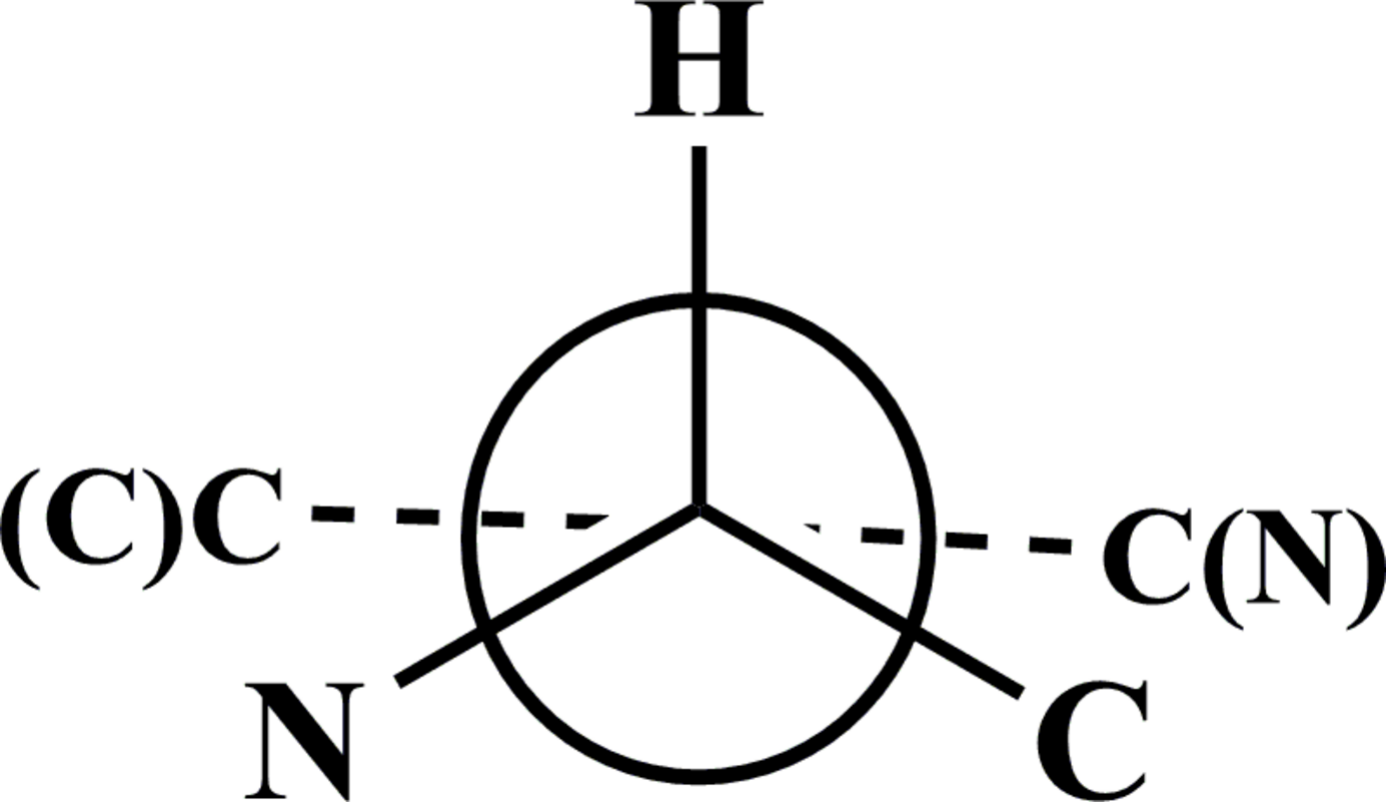
The spatial arrangement of the pyridine and piperidine rings in *N*-aryl­sulfonyl anabasine (**1**–**4**)

In studies by Wojciechowska-Nowak *et al.* on the structures of salts of anabasine derivatives, four conformations were proposed based on the arrangement of the piperidine and pyridine rings. The N-aryl­sulfonyl anabasine derivatives **1**–**4** studied here differ from the proposed conformations (Fig. 2 ▸).

The position of the aryl­sulfonyl group relative to the piperidine ring in structures (around the N1—S1 and S1—C7 bonds) is nearly identical. The mutual arrangement of the piperidine and benzene rings was analyzed using the C2′—N1′—S1—O1 and O1—S1—C7—C12 torsion angles, which are 34.8 (2) and −24.4 (3)° for **1**, 33.7 (2) and −31.5 (3)° for **2**, 37.2 (4) and −24.0 (4)° for **3**, and 35.6 (3) and −32.3 (3)° for **4**, respectively. In all structures, such an arrangement of groups can be explained by the presence of intra­molecular hydrogen bonding.

The mutual arrangement of planar aromatic rings relative to the six-membered saturated heterocycle in compounds **2**–**4** is nearly identical. The nature of inter­molecular van der Waals contacts in these structures is also similar, indicating the formation of isostructural crystals in the packing (space group *P*2_1_2_1_2_1_) with identical unit-cell parameters (Table 5).

It was established that in the crystal structures, the similar arrangement of the piperidine ring and the benzene ring connected via the sulfonyl group is due to inter­molecular inter­actions. Substituents on the benzene ring (Cl, CH_3_, OCH_3_) do not significantly affect the mutual arrangement of the rings.

## 3. Supra­molecular features

**Figure 3:**
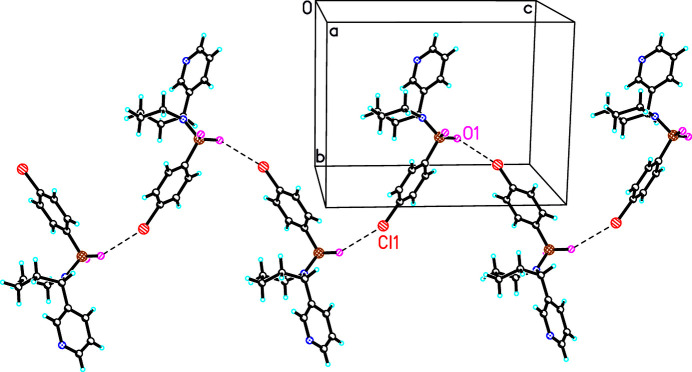
Observed inter­molecular O1⋯Cl1 contacts in the crystal structure of **1** (the mol­ecules are linked along the *c*-axis direction).

The analysis of inter­molecular inter­actions in all studied structures revealed only weak hydrogen bonding, which plays a key role in consolidating the crystal packing (Tables 1 ▸–4 ▸ ▸ ▸, Figs. 3 ▸–6 ▸ ▸ ▸).

A consistent intra­molecular C—H⋯O hydrogen bond was observed to consolidate the mol­ecular conformation in the solid state. This inter­action involves the piperidine ring via the asymmetric carbon atom and the oxygen atom of the SO_2_ group (C2′—H2*’A*⋯O1).

In the crystal structure of compound **1**, an infinite ribbon is formed along the *c-*axis direction, driven by donor–acceptor inter­actions between the oxygen atom of the SO_2_ group and the chlorine atom at the C10 position [Cl⋯O distance = 3.127 (2) Å; symmetry operation: 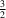 − *x*, 2 − *y*, −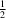 + *z*; Fig. 3 ▸). These ribbons are further inter­connected along the *a*-axis direction through inter­molecular C12—H12⋯O2 hydrogen bonds. Additionally, the chlorine atom participates in weak C–H⋯Cl (C3′—H3′*B*⋯Cl1 and C8—H8⋯Cl1) hydrogen bonding inter­actions.

**Figure 4:**
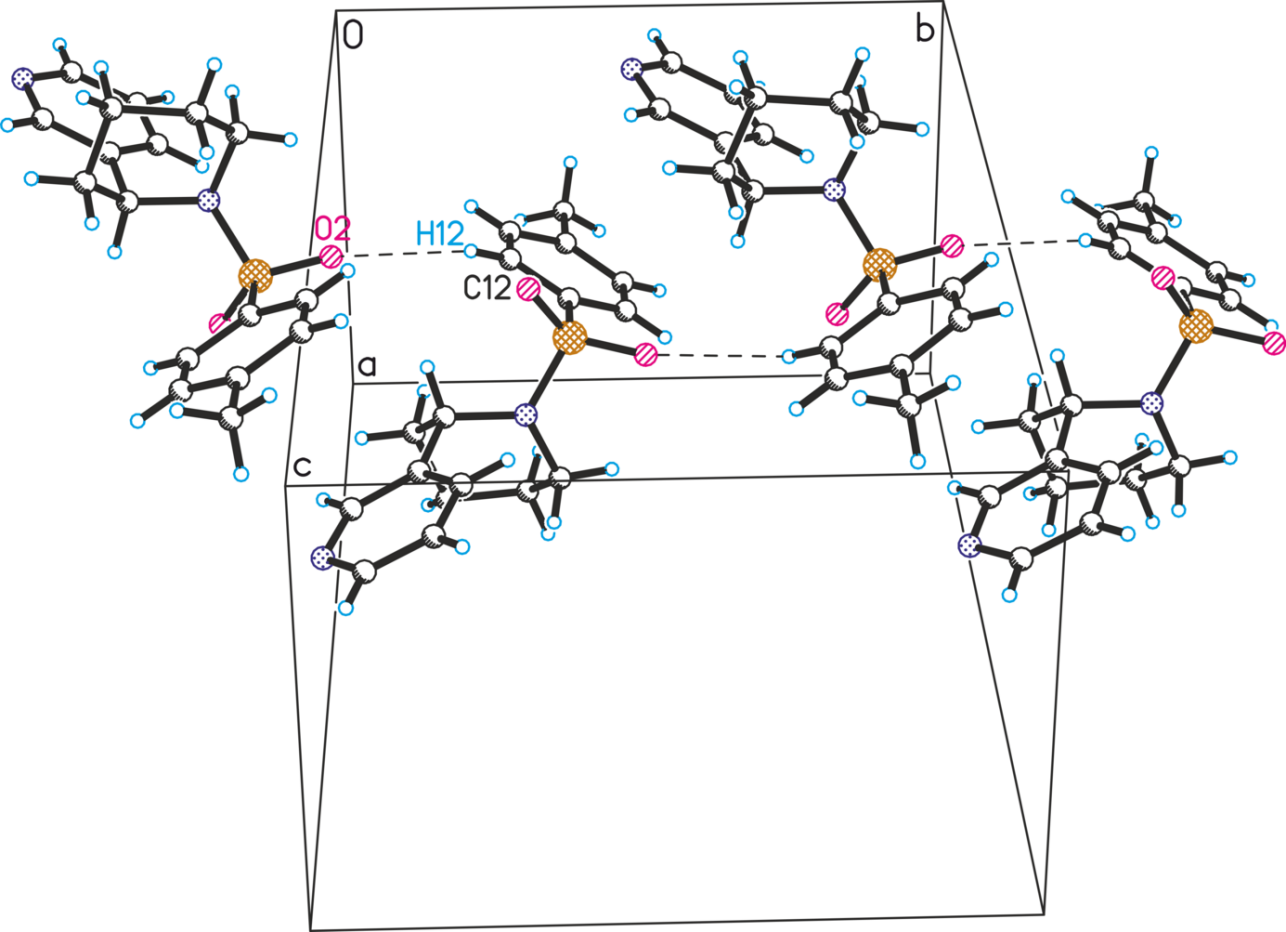
Observed inter­molecular C12—H12⋯O2 inter­actions in the crystal structure of **2** (the mol­ecules are linked along the *b*-axis direction).

**Figure 6:**
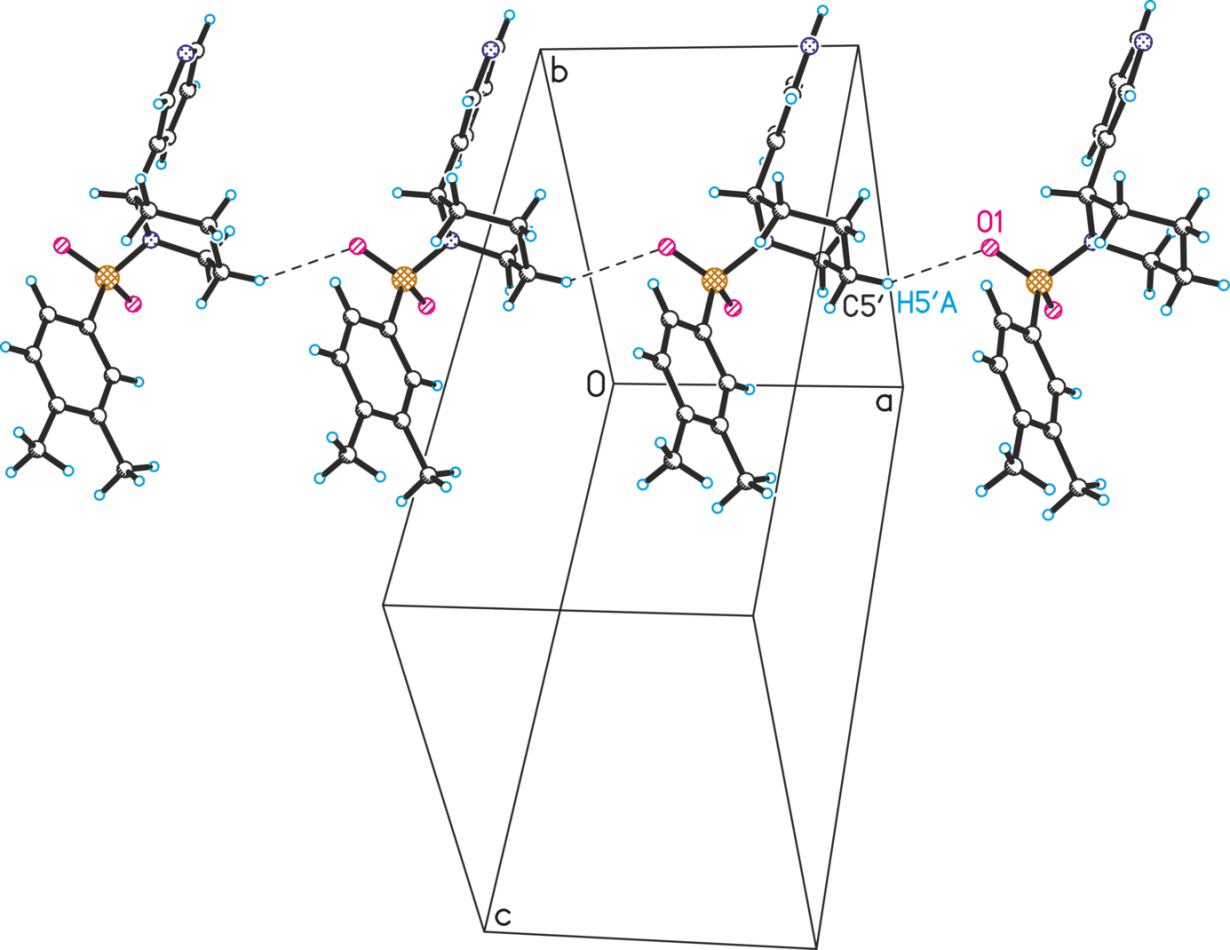
Observed inter­molecular C5′—H5′*A*⋯O1 inter­actions in the crystal structure of **4** (the mol­ecules are linked along the *a*-axis direction).

In the crystal structures of compounds **2** and **3**, similar inter­molecular hydrogen bonding patterns are observed, where mol­ecules are linked along the *b*-axis direction via C12—H12⋯O2 inter­actions (Figs. 4 ▸ and 5 ▸). The resulting chains are further consolidated by C5′—H5′*A*⋯O1 hydrogen bonds. In the crystal structure of **4**, this type of inter­action leads to the formation of mol­ecular chains along the *a*-axis direction (Fig. 6 ▸). In contrast to the previous structures, the chains in structure **4** are inter­connected via inter­molecular C5—H5⋯O1 hydrogen bonds.

## 4. Database survey

A search of anabasine derivatives in the Cambridge Structural Database (CSD, updated to November 2025; Groom *et al.*, 2016 ▸) yielded 37 results. Of them, 13 are N-derivatives of anabasin alkaloids.

The axial orientation of the pyridine ring is observed in the crystal structures of *N*-ethyl-2-(pyridin-3-yl)piperidine-1-carbo­­thio­amide (ATUGAZ; Nurkenov *et al.*, 2016 ▸) and 2-(pyridin-3-yl)-*N*-[2-(vin­yloxy)eth­yl]piperidine-1-carbothi­amide (QELPON; Ibraev *et al.*, 2006 ▸).

Structurally similar compounds in terms of the orientation of the pyridine ring relative to the piperidine ring and the distance between the nitro­gen atoms are *N*-(anabasinyl-1-carbono­thio­yl)-2-furamide (FUSKUA; Kulakov *et al.*, 2009 ▸) and 2-(pyridin-3-yl)-*N*-[2-(vin­yloxy)eth­yl]piperidine-1-carbo­thi­amide (QELPON; Ibraev *et al.*, 2006 ▸).

## 5. Synthesis and crystallization

(*S*)-3-{1-[(4-Chloro­phen­yl)sulfon­yl]piperidin-2-yl}pyridine (**1**), (*S*)-3-[1-(4-methyl­phen­yl)piperidin-2-yl]pyridine (**2**), (*S*)-3-{1-[(4-meth­oxy­phen­yl)sulfon­yl]piperidin-2-yl}pyridine (**3**), and (*S*)-3-{1-[(3,4-di­methyl­phen­yl)sulfon­yl]piperidin-2-yl}pyridine (**4**) were synthesized according to the reported method (Olimova *et al.*, 2025 ▸). Colourless single crystals of the compounds, suitable for X-ray diffraction analysis, were successfully obtained from ethanol.

## 6. Refinement

Crystal data, data collection and structure refinement details are summarized in Table 5 ▸. All hydrogen atoms were found in difference maps and then freely refined with isotropic shift parameters, resulting in C—H distances of 0.97 Å for CH_2_, 0.96 Å for CH_3_ and 0.93 Å for C_ar_.

## Supplementary Material

Crystal structure: contains datablock(s) 1, 2, 3, 4, manuscript. DOI: 10.1107/S2056989026004524/dx2068sup1.cif

Structure factors: contains datablock(s) 1. DOI: 10.1107/S2056989026004524/dx20681sup2.hkl

Structure factors: contains datablock(s) 2. DOI: 10.1107/S2056989026004524/dx20682sup3.hkl

Structure factors: contains datablock(s) 3. DOI: 10.1107/S2056989026004524/dx20683sup4.hkl

Structure factors: contains datablock(s) 4. DOI: 10.1107/S2056989026004524/dx20684sup5.hkl

Supporting information file. DOI: 10.1107/S2056989026004524/dx20681sup6.cml

Supporting information file. DOI: 10.1107/S2056989026004524/dx20682sup7.cml

Supporting information file. DOI: 10.1107/S2056989026004524/dx20683sup8.cml

Supporting information file. DOI: 10.1107/S2056989026004524/dx20684sup9.cml

CCDC references: 2550374, 2550373, 2550372, 2550371

Additional supporting information:  crystallographic information; 3D view; checkCIF report

## Figures and Tables

**Figure 5:**
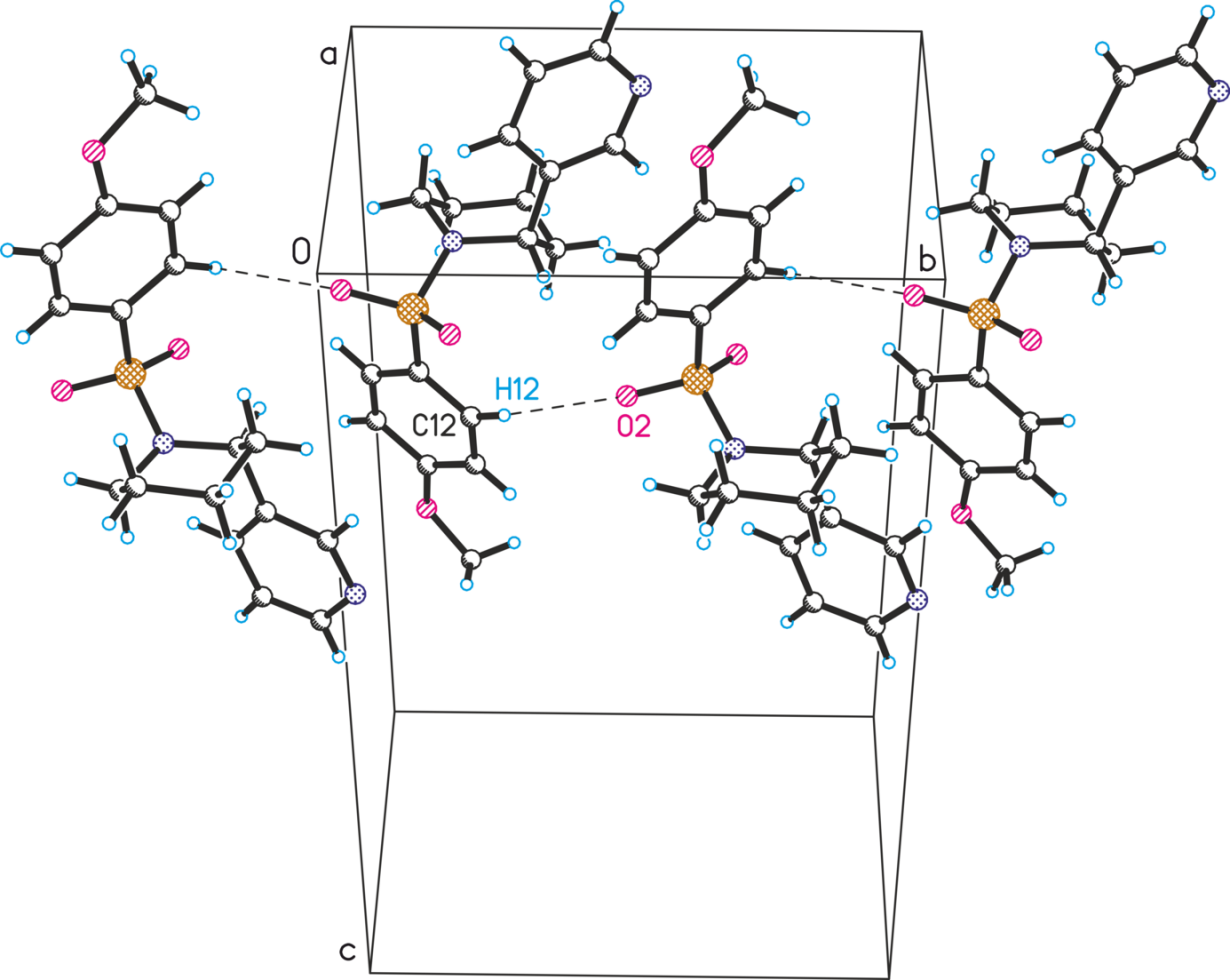
Observed inter­molecular C12—H12⋯O2 inter­actions in the crystal structure of **3** (the mol­ecules are linked along the *b*-axis direction).

**Table 1:** Hydrogen-bond geometry (Å, °) for **1**[Chem scheme1]

*D*—H⋯*A*	*D*—H	H⋯*A*	*D*⋯*A*	*D*—H⋯*A*
C12—H12⋯O2^i^	0.93	2.58	3.383 (4)	145
C3′—H3′*B*⋯Cl1^ii^	0.97	2.98	3.923 (4)	163
C8—H8⋯Cl1^iii^	0.93	2.97	3.807 (3)	150
C2′—H2′*A*⋯O1	0.98	2.38	2.880 (4)	111

Symmetry codes: (i) 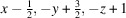; (ii) 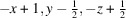; (iii) 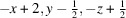.

**Table 2:** Hydrogen-bond geometry (Å, °) for **2**[Chem scheme1]

*D*—H⋯*A*	*D*—H	H⋯*A*	*D*⋯*A*	*D*—H⋯*A*
C12—H12⋯O2^i^	0.93	2.62	3.474 (4)	152
C5′—H5′*A*⋯O1^ii^	0.97	2.51	3.369 (4)	147
C2′—H2′*A*⋯O1	0.98	2.37	2.884 (4)	112

Symmetry codes: (i) 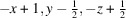; (ii) 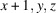.

**Table 3:** Hydrogen-bond geometry (Å, °) for **3**[Chem scheme1]

*D*—H⋯*A*	*D*—H	H⋯*A*	*D*⋯*A*	*D*—H⋯*A*
C12—H12⋯O2^i^	0.93	2.47	3.252 (5)	142
C5′—H5′*A*⋯O1^ii^	0.97	2.49	3.298 (5)	140
C2′—H2′*A*⋯O1	0.98	2.37	2.880 (5)	111

Symmetry codes: (i) 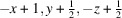; (ii) 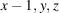.

**Table 4:** Hydrogen-bond geometry (Å, °) for **4**[Chem scheme1]

*D*—H⋯*A*	*D*—H	H⋯*A*	*D*⋯*A*	*D*—H⋯*A*
C5′—H5′*A*⋯O1^i^	0.97	2.60	3.462 (5)	148
C5—H5⋯O1^ii^	0.93	2.64	3.384 (5)	138
C2′—H2′*A*⋯O1	0.98	2.39	2.892 (4)	111

Symmetry codes: (i) 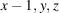; (ii) 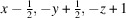.

**Table 5:** Experimental details

	**1**	**2**	**3**	**4**
Crystal data				
Chemical formula	C_16_H_17_ClN_2_O_2_S	C_17_H_20_N_2_O_2_S	C_17_H_20_N_2_O_3_S	C_18_H_22_N_2_O_2_S
*M*_r_	336.83	316.41	332.41	330.43
Crystal system, space group	Orthorhombic, *P*2_1_2_1_2_1_	Orthorhombic, *P*2_1_2_1_2_1_	Orthorhombic, *P*2_1_2_1_2_1_	Orthorhombic, *P*2_1_2_1_2_1_
Temperature (K)	294	297	297	295
*a*, *b*, *c* (Å)	10.694 (2), 10.715 (2), 14.110 (3)	7.8933 (16), 11.408 (2), 18.195 (4)	7.9758 (16), 11.131 (2), 18.754 (4)	7.9087 (16), 11.737 (2), 18.117 (4)
*V* (Å^3^)	1616.8 (6)	1638.5 (6)	1664.9 (6)	1681.6 (6)
*Z*	4	4	4	4
Radiation type	Cu *K*α	Cu *K*α	Cu *K*α	Cu *K*α
μ (mm^−1^)	3.37	1.82	1.87	1.80
Crystal size (mm)	0.50 × 0.45 × 0.30	0.45 × 0.30 × 0.25	0.30 × 0.25 × 0.25	0.45 × 0.25 × 0.22

Data collection				
Diffractometer	Bruker D8 VENTURE dual wavelength Mo/Cu	Xcalibur, Ruby	Xcalibur, Ruby	Xcalibur, Ruby
Absorption correction	Multi-scan (*SADABS*; Krause *et al.*, 2015 ▸)	Multi-scan (*SADABS*; Krause *et al.*, 2015 ▸)	Multi-scan (*SADABS*; Krause *et al.*, 2015 ▸)	Multi-scan (*SADABS*; Krause *et al.*, 2015 ▸)
*T*_min_, *T*_max_	0.32, 0.43	0.79, 1.00	0.78, 1.00	0.88, 1.00
No. of measured, independent and observed [*I* > 2σ(*I*)] reflections	50181, 3234, 3222	11987, 3372, 3194	12142, 3427, 2899	12220, 3428, 2886
*R*_int_	0.029	0.033	0.048	0.045
(sin θ/λ)_max_ (Å^−1^)	0.625	0.630	0.629	0.630

Refinement				
*R*[*F*^2^ > 2σ(*F*^2^)], *wR*(*F*^2^), *S*	0.039, 0.102, 1.06	0.051, 0.123, 1.12	0.054, 0.133, 1.10	0.045, 0.111, 1.07
No. of reflections	3234	3372	3427	3428
No. of parameters	199	200	209	210
H-atom treatment	H-atom parameters constrained	H-atom parameters constrained	H-atom parameters constrained	H-atom parameters constrained
Δρ_max_, Δρ_min_ (e Å^−3^)	0.30, −0.44	0.33, −0.63	0.23, −0.53	0.21, −0.34
Absolute structure	Flack *x* determined using 1346 quotients [(*I*^+^)−(*I*^−^)]/[(*I*^+^)+(*I*^−^)] (Parsons *et al.*, 2013 ▸)	Flack *x* determined using 1275 quotients [(*I*^+^)−(*I*^−^)]/[(*I*^+^)+(*I*^−^)] (Parsons *et al.*, 2013 ▸)	Flack *x* determined using 1030 quotients [(*I*^+^)−(*I*^−^)]/[(*I*^+^)+(*I*^−^)] (Parsons *et al.*, 2013 ▸)	Flack *x* determined using 1007 quotients [(*I*^+^)−(*I*^−^)]/[(*I*^+^)+(*I*^−^)] (Parsons *et al.*, 2013 ▸)
Absolute structure parameter	0.094 (3)	0.005 (8)	−0.014 (15)	0.011 (14)

Computer programs: *APEX5* and *SAINT* (Bruker, 2012 ▸), *CrysAlis PRO* (Rigaku OD, 2021 ▸), *SHELXT2018/2* (Sheldrick, 2015*a* ▸), *SHELXL2019/3* (Sheldrick, 2015*b* ▸), *XP* in *SHELXTL* (Sheldrick, 2008 ▸), *Mercury* (Macrae *et al.*, 2020 ▸), *PLATON* (Spek, 2020 ▸) and *publCIF* (Westrip, 2010 ▸).

## supplementary crystallographic information

### (S)-3-{1-[(4-Chlorophenyl)sulfonyl]piperidin-2-yl}pyridine (1) Crystal data

**Table:**

C_16_H_17_ClN_2_O_2_S	*D*_x_ = 1.384 Mg m^−^^3^
*M**_r_* = 336.83	Cu *K*α radiation, λ = 1.54178 Å
Orthorhombic, *P*2_1_2_1_2_1_	Cell parameters from 9773 reflections
*a* = 10.694 (2) Å	θ = 4.1–74.2°
*b* = 10.715 (2) Å	µ = 3.37 mm^−^^1^
*c* = 14.110 (3) Å	*T* = 294 K
*V* = 1616.8 (6) Å^3^	Prism, colourless
*Z* = 4	0.50 × 0.45 × 0.30 mm
*F*(000) = 704	

### (S)-3-{1-[(4-Chlorophenyl)sulfonyl]piperidin-2-yl}pyridine (1) Data collection

**Table:**

Bruker D8 VENTURE dual wavelength Mo/Cu diffractometer	3234 independent reflections
Radiation source: microfocus sealed X-ray tube, INCOATEC IµS	3222 reflections with *I* > 2σ(*I*)
Detector resolution: 7.39 pixels mm^-1^	*R*_int_ = 0.029
φ and ω scans	θ_max_ = 74.4°, θ_min_ = 5.2°
Absorption correction: multi-scan (SADABS; Krause *et al.*, 2015)	*h* = −13→13
*T*_min_ = 0.32, *T*_max_ = 0.43	*k* = −13→13
50181 measured reflections	*l* = −17→17

### (S)-3-{1-[(4-Chlorophenyl)sulfonyl]piperidin-2-yl}pyridine (1) Refinement

**Table:**

Refinement on *F*^2^	Secondary atom site location: dual
Least-squares matrix: full	Hydrogen site location: inferred from neighbouring sites
*R*[*F*^2^ > 2σ(*F*^2^)] = 0.039	H-atom parameters constrained
*wR*(*F*^2^) = 0.102	*w* = 1/[σ^2^(*F*_o_^2^) + (0.0574*P*)^2^ + 0.4484*P*] where *P* = (*F*_o_^2^ + 2*F*_c_^2^)/3
*S* = 1.06	(Δ/σ)_max_ < 0.001
3234 reflections	Δρ_max_ = 0.30 e Å^−^^3^
199 parameters	Δρ_min_ = −0.44 e Å^−^^3^
0 restraints	Absolute structure: Flack *x* determined using 1346 quotients [(*I*^+^)-(*I*^-^)]/[(*I*^+^)+(*I*^-^)] (Parsons *et al.*, 2013)
Primary atom site location: dual	Absolute structure parameter: 0.094 (3)

### (S)-3-{1-[(4-Chlorophenyl)sulfonyl]piperidin-2-yl}pyridine (1) Special details

**Table:**

Geometry. All esds (except the esd in the dihedral angle between two l.s. planes) are estimated using the full covariance matrix. The cell esds are taken into account individually in the estimation of esds in distances, angles and torsion angles; correlations between esds in cell parameters are only used when they are defined by crystal symmetry. An approximate (isotropic) treatment of cell esds is used for estimating esds involving l.s. planes.

### (S)-3-{1-[(4-Chlorophenyl)sulfonyl]piperidin-2-yl}pyridine (1) Fractional atomic coordinates and isotropic or equivalent isotropic displacement parameters (Å^2^)

**Table:**

	*x*	*y*	*z*	*U*_iso_*/*U*_eq_	
S1	0.78885 (6)	0.66179 (6)	0.48625 (4)	0.04518 (19)	
Cl1	0.82268 (11)	1.15407 (11)	0.25168 (8)	0.0926 (4)	
O1	0.7158 (3)	0.6849 (2)	0.56915 (13)	0.0642 (6)	
O2	0.9117 (2)	0.6108 (2)	0.4948 (2)	0.0731 (7)	
N1	0.3482 (4)	0.3020 (4)	0.4288 (3)	0.0849 (11)	
N1'	0.7089 (2)	0.5681 (2)	0.41990 (15)	0.0406 (5)	
C2	0.4014 (3)	0.4113 (4)	0.4065 (3)	0.0643 (9)	
H2	0.359882	0.463138	0.364013	0.077*	
C3	0.5152 (3)	0.4518 (3)	0.4432 (2)	0.0457 (6)	
C4	0.5723 (4)	0.3748 (3)	0.5077 (2)	0.0620 (8)	
H4	0.646868	0.398968	0.536173	0.074*	
C5	0.5184 (5)	0.2607 (4)	0.5303 (3)	0.0776 (11)	
H5	0.557477	0.206614	0.572456	0.093*	
C6	0.4083 (5)	0.2297 (4)	0.4899 (4)	0.0817 (13)	
H6	0.372584	0.153400	0.505841	0.098*	
C2'	0.5711 (2)	0.5783 (3)	0.4172 (2)	0.0450 (6)	
H2'A	0.545691	0.638507	0.465762	0.054*	
C3'	0.5304 (4)	0.6293 (4)	0.3211 (3)	0.0808 (13)	
H3'A	0.557074	0.715537	0.315568	0.097*	
H3'B	0.439910	0.627501	0.317113	0.097*	
C4'	0.5852 (5)	0.5542 (6)	0.2392 (3)	0.0984 (18)	
H4'A	0.554452	0.469097	0.241802	0.118*	
H4'B	0.559108	0.590475	0.179400	0.118*	
C5'	0.7247 (5)	0.5543 (5)	0.2453 (3)	0.0894 (14)	
H5'A	0.755575	0.638741	0.237147	0.107*	
H5'B	0.758889	0.503317	0.194713	0.107*	
C6'	0.7677 (3)	0.5041 (3)	0.3396 (2)	0.0599 (8)	
H6'A	0.748388	0.415765	0.342963	0.072*	
H6'B	0.857766	0.513171	0.344197	0.072*	
C7	0.8030 (2)	0.8051 (2)	0.42494 (19)	0.0424 (5)	
C8	0.9004 (3)	0.8207 (3)	0.3612 (3)	0.0574 (8)	
H8	0.960987	0.759082	0.354115	0.069*	
C9	0.9062 (4)	0.9289 (4)	0.3083 (3)	0.0680 (9)	
H9	0.970115	0.940354	0.264489	0.082*	
C10	0.8171 (3)	1.0196 (3)	0.3209 (2)	0.0587 (8)	
C11	0.7225 (3)	1.0061 (3)	0.3849 (3)	0.0596 (7)	
H11	0.663730	1.069156	0.393154	0.071*	
C12	0.7150 (3)	0.8969 (3)	0.4374 (2)	0.0522 (6)	
H12	0.650597	0.885872	0.480863	0.063*	

### (S)-3-{1-[(4-Chlorophenyl)sulfonyl]piperidin-2-yl}pyridine (1) Atomic displacement parameters (Å^2^)

**Table:**

	*U*^11^	*U*^22^	*U*^33^	*U*^12^	*U*^13^	*U*^23^
S1	0.0438 (3)	0.0471 (3)	0.0446 (3)	−0.0057 (3)	−0.0095 (3)	0.0027 (3)
Cl1	0.0952 (8)	0.0797 (6)	0.1027 (8)	−0.0302 (6)	−0.0210 (6)	0.0445 (6)
O1	0.0915 (16)	0.0653 (13)	0.0358 (9)	−0.0170 (13)	0.0048 (11)	−0.0063 (9)
O2	0.0457 (11)	0.0698 (14)	0.104 (2)	0.0008 (10)	−0.0306 (13)	0.0130 (14)
N1	0.080 (2)	0.079 (2)	0.096 (2)	−0.0393 (18)	0.008 (2)	−0.007 (2)
N1'	0.0352 (10)	0.0437 (11)	0.0430 (10)	−0.0005 (9)	0.0010 (9)	−0.0058 (9)
C2	0.0530 (17)	0.067 (2)	0.073 (2)	−0.0170 (16)	−0.0035 (16)	0.0014 (17)
C3	0.0430 (13)	0.0475 (14)	0.0467 (13)	−0.0068 (11)	0.0072 (11)	−0.0054 (11)
C4	0.0677 (19)	0.0587 (17)	0.0595 (18)	−0.0078 (14)	0.0022 (15)	0.0091 (15)
C5	0.099 (3)	0.062 (2)	0.072 (2)	−0.007 (2)	0.017 (2)	0.0197 (19)
C6	0.102 (3)	0.061 (2)	0.082 (3)	−0.027 (2)	0.030 (2)	−0.001 (2)
C2'	0.0352 (12)	0.0430 (13)	0.0568 (15)	−0.0002 (10)	−0.0013 (11)	0.0011 (12)
C3'	0.060 (2)	0.076 (2)	0.106 (3)	−0.0196 (18)	−0.034 (2)	0.042 (2)
C4'	0.114 (4)	0.135 (4)	0.0460 (18)	−0.056 (3)	−0.025 (2)	0.022 (2)
C5'	0.115 (3)	0.107 (3)	0.0457 (17)	−0.044 (3)	0.015 (2)	−0.0083 (19)
C6'	0.0606 (19)	0.0631 (18)	0.0559 (16)	−0.0027 (15)	0.0172 (14)	−0.0162 (14)
C7	0.0364 (12)	0.0451 (12)	0.0457 (12)	−0.0089 (10)	−0.0044 (10)	0.0014 (10)
C8	0.0390 (13)	0.0601 (18)	0.0731 (19)	−0.0042 (13)	0.0102 (13)	0.0010 (15)
C9	0.0545 (18)	0.078 (2)	0.071 (2)	−0.0210 (17)	0.0160 (16)	0.0097 (18)
C10	0.0556 (18)	0.0560 (16)	0.0646 (18)	−0.0188 (14)	−0.0127 (14)	0.0121 (14)
C11	0.0514 (17)	0.0506 (15)	0.077 (2)	0.0018 (14)	−0.0025 (16)	0.0047 (15)
C12	0.0436 (13)	0.0533 (15)	0.0597 (16)	−0.0031 (12)	0.0055 (13)	−0.0002 (12)

### (S)-3-{1-[(4-Chlorophenyl)sulfonyl]piperidin-2-yl}pyridine (1) Geometric parameters (Å, º)

**Table:**

S1—O2	1.428 (2)	C3'—H3'A	0.9700
S1—O1	1.428 (2)	C3'—H3'B	0.9700
S1—N1'	1.617 (2)	C4'—C5'	1.494 (8)
S1—C7	1.769 (3)	C4'—H4'A	0.9700
Cl1—C10	1.741 (3)	C4'—H4'B	0.9700
N1—C6	1.326 (7)	C5'—C6'	1.507 (6)
N1—C2	1.339 (5)	C5'—H5'A	0.9700
N1'—C6'	1.466 (3)	C5'—H5'B	0.9700
N1'—C2'	1.478 (3)	C6'—H6'A	0.9700
C2—C3	1.392 (4)	C6'—H6'B	0.9700
C2—H2	0.9300	C7—C12	1.372 (4)
C3—C4	1.371 (5)	C7—C8	1.387 (4)
C3—C2'	1.526 (4)	C8—C9	1.380 (5)
C4—C5	1.389 (5)	C8—H8	0.9300
C4—H4	0.9300	C9—C10	1.373 (6)
C5—C6	1.350 (7)	C9—H9	0.9300
C5—H5	0.9300	C10—C11	1.364 (5)
C6—H6	0.9300	C11—C12	1.387 (4)
C2'—C3'	1.525 (5)	C11—H11	0.9300
C2'—H2'A	0.9800	C12—H12	0.9300
C3'—C4'	1.525 (8)		
			
O2—S1—O1	120.04 (18)	C5'—C4'—C3'	109.8 (3)
O2—S1—N1'	107.32 (14)	C5'—C4'—H4'A	109.7
O1—S1—N1'	107.00 (14)	C3'—C4'—H4'A	109.7
O2—S1—C7	107.15 (14)	C5'—C4'—H4'B	109.7
O1—S1—C7	107.28 (14)	C3'—C4'—H4'B	109.7
N1'—S1—C7	107.51 (12)	H4'A—C4'—H4'B	108.2
C6—N1—C2	117.2 (4)	C4'—C5'—C6'	110.8 (3)
C6'—N1'—C2'	116.2 (2)	C4'—C5'—H5'A	109.5
C6'—N1'—S1	120.7 (2)	C6'—C5'—H5'A	109.5
C2'—N1'—S1	119.75 (18)	C4'—C5'—H5'B	109.5
N1—C2—C3	123.8 (4)	C6'—C5'—H5'B	109.5
N1—C2—H2	118.1	H5'A—C5'—H5'B	108.1
C3—C2—H2	118.1	N1'—C6'—C5'	112.6 (3)
C4—C3—C2	116.6 (3)	N1'—C6'—H6'A	109.1
C4—C3—C2'	121.3 (3)	C5'—C6'—H6'A	109.1
C2—C3—C2'	122.0 (3)	N1'—C6'—H6'B	109.1
C3—C4—C5	119.8 (4)	C5'—C6'—H6'B	109.1
C3—C4—H4	120.1	H6'A—C6'—H6'B	107.8
C5—C4—H4	120.1	C12—C7—C8	120.8 (3)
C6—C5—C4	118.8 (4)	C12—C7—S1	120.1 (2)
C6—C5—H5	120.6	C8—C7—S1	119.1 (2)
C4—C5—H5	120.6	C9—C8—C7	119.0 (3)
N1—C6—C5	123.7 (4)	C9—C8—H8	120.5
N1—C6—H6	118.2	C7—C8—H8	120.5
C5—C6—H6	118.2	C10—C9—C8	119.6 (3)
N1'—C2'—C3'	109.5 (3)	C10—C9—H9	120.2
N1'—C2'—C3	108.6 (2)	C8—C9—H9	120.2
C3'—C2'—C3	114.9 (3)	C11—C10—C9	121.7 (3)
N1'—C2'—H2'A	107.9	C11—C10—Cl1	119.0 (3)
C3'—C2'—H2'A	107.9	C9—C10—Cl1	119.3 (3)
C3—C2'—H2'A	107.9	C10—C11—C12	119.1 (3)
C2'—C3'—C4'	112.0 (3)	C10—C11—H11	120.5
C2'—C3'—H3'A	109.2	C12—C11—H11	120.5
C4'—C3'—H3'A	109.2	C7—C12—C11	119.8 (3)
C2'—C3'—H3'B	109.2	C7—C12—H12	120.1
C4'—C3'—H3'B	109.2	C11—C12—H12	120.1
H3'A—C3'—H3'B	107.9		
			
O2—S1—N1'—C6'	−36.4 (3)	C3—C2'—C3'—C4'	−69.6 (4)
O1—S1—N1'—C6'	−166.5 (2)	C2'—C3'—C4'—C5'	−57.9 (5)
C7—S1—N1'—C6'	78.6 (3)	C3'—C4'—C5'—C6'	56.6 (5)
O2—S1—N1'—C2'	164.8 (2)	C2'—N1'—C6'—C5'	50.8 (4)
O1—S1—N1'—C2'	34.8 (2)	S1—N1'—C6'—C5'	−108.7 (3)
C7—S1—N1'—C2'	−80.2 (2)	C4'—C5'—C6'—N1'	−52.9 (5)
C6—N1—C2—C3	0.4 (6)	O2—S1—C7—C12	−154.5 (2)
N1—C2—C3—C4	−1.8 (5)	O1—S1—C7—C12	−24.4 (3)
N1—C2—C3—C2'	−178.9 (3)	N1'—S1—C7—C12	90.4 (2)
C2—C3—C4—C5	2.4 (5)	O2—S1—C7—C8	28.5 (3)
C2'—C3—C4—C5	179.6 (3)	O1—S1—C7—C8	158.6 (2)
C3—C4—C5—C6	−1.9 (6)	N1'—S1—C7—C8	−86.6 (3)
C2—N1—C6—C5	0.2 (7)	C12—C7—C8—C9	−1.6 (5)
C4—C5—C6—N1	0.5 (7)	S1—C7—C8—C9	175.4 (3)
C6'—N1'—C2'—C3'	−49.9 (4)	C7—C8—C9—C10	1.1 (5)
S1—N1'—C2'—C3'	109.8 (3)	C8—C9—C10—C11	0.3 (5)
C6'—N1'—C2'—C3	76.3 (3)	C8—C9—C10—Cl1	−178.3 (3)
S1—N1'—C2'—C3	−124.0 (2)	C9—C10—C11—C12	−1.1 (5)
C4—C3—C2'—N1'	34.0 (4)	Cl1—C10—C11—C12	177.4 (3)
C2—C3—C2'—N1'	−149.0 (3)	C8—C7—C12—C11	0.8 (5)
C4—C3—C2'—C3'	157.0 (3)	S1—C7—C12—C11	−176.2 (2)
C2—C3—C2'—C3'	−26.0 (4)	C10—C11—C12—C7	0.6 (5)
N1'—C2'—C3'—C4'	52.9 (4)		

### (S)-3-{1-[(4-Chlorophenyl)sulfonyl]piperidin-2-yl}pyridine (1) Hydrogen-bond geometry (Å, º)

**Table:**

*D*—H···*A*	*D*—H	H···*A*	*D*···*A*	*D*—H···*A*
C12—H12···O2^i^	0.93	2.58	3.383 (4)	145
C3′—H3′*B*···Cl1^ii^	0.97	2.98	3.923 (4)	163
C8—H8···Cl1^iii^	0.93	2.97	3.807 (3)	150
C2′—H2′*A*···O1	0.98	2.38	2.880 (4)	111

Symmetry codes: (i) *x*−1/2, −*y*+3/2, −*z*+1; (ii) −*x*+1, *y*−1/2, −*z*+1/2; (iii) −*x*+2, *y*−1/2, −*z*+1/2.

### (S)-3-[1-(4-Methylphenyl)piperidin-2-yl]pyridine (2) Crystal data

**Table:**

C_17_H_20_N_2_O_2_S	*D*_x_ = 1.283 Mg m^−^^3^
*M**_r_* = 316.41	Cu *K*α radiation, λ = 1.54184 Å
Orthorhombic, *P*2_1_2_1_2_1_	Cell parameters from 7339 reflections
*a* = 7.8933 (16) Å	θ = 4.6–75.5°
*b* = 11.408 (2) Å	µ = 1.82 mm^−^^1^
*c* = 18.195 (4) Å	*T* = 297 K
*V* = 1638.5 (6) Å^3^	Prism, colourless
*Z* = 4	0.45 × 0.30 × 0.25 mm
*F*(000) = 672	

### (S)-3-[1-(4-Methylphenyl)piperidin-2-yl]pyridine (2) Data collection

**Table:**

Xcalibur, Ruby diffractometer	3372 independent reflections
Radiation source: Enhance (Cu) X-ray Source	3194 reflections with *I* > 2σ(*I*)
Detector resolution: 10.25 pixels mm^-1^	*R*_int_ = 0.033
ω scans	θ_max_ = 76.1°, θ_min_ = 4.6°
Absorption correction: multi-scan (SADABS; Krause *et al.*, 2015)	*h* = −6→9
*T*_min_ = 0.79, *T*_max_ = 1.00	*k* = −13→14
11987 measured reflections	*l* = −22→22

### (S)-3-[1-(4-Methylphenyl)piperidin-2-yl]pyridine (2) Refinement

**Table:**

Refinement on *F*^2^	Secondary atom site location: dual
Least-squares matrix: full	Hydrogen site location: inferred from neighbouring sites
*R*[*F*^2^ > 2σ(*F*^2^)] = 0.051	H-atom parameters constrained
*wR*(*F*^2^) = 0.123	*w* = 1/[σ^2^(*F*_o_^2^) + (0.0837*P*)^2^ + 0.0529*P*] where *P* = (*F*_o_^2^ + 2*F*_c_^2^)/3
*S* = 1.12	(Δ/σ)_max_ < 0.001
3372 reflections	Δρ_max_ = 0.33 e Å^−^^3^
200 parameters	Δρ_min_ = −0.63 e Å^−^^3^
0 restraints	Absolute structure: Flack *x* determined using 1275 quotients [(*I*^+^)-(*I*^-^)]/[(*I*^+^)+(*I*^-^)] (Parsons *et al.*, 2013)
Primary atom site location: dual	Absolute structure parameter: 0.005 (8)

### (S)-3-[1-(4-Methylphenyl)piperidin-2-yl]pyridine (2) Special details

**Table:**

Geometry. All esds (except the esd in the dihedral angle between two l.s. planes) are estimated using the full covariance matrix. The cell esds are taken into account individually in the estimation of esds in distances, angles and torsion angles; correlations between esds in cell parameters are only used when they are defined by crystal symmetry. An approximate (isotropic) treatment of cell esds is used for estimating esds involving l.s. planes.

### (S)-3-[1-(4-Methylphenyl)piperidin-2-yl]pyridine (2) Fractional atomic coordinates and isotropic or equivalent isotropic displacement parameters (Å^2^)

**Table:**

	*x*	*y*	*z*	*U*_iso_*/*U*_eq_	
S1	0.55288 (8)	0.37284 (6)	0.27853 (4)	0.0502 (2)	
O1	0.4044 (3)	0.3086 (3)	0.29892 (14)	0.0721 (7)	
O2	0.5731 (4)	0.4921 (2)	0.30207 (14)	0.0760 (7)	
N1	0.8626 (7)	−0.0151 (4)	0.4744 (2)	0.1120 (15)	
N1'	0.7130 (3)	0.30057 (18)	0.31083 (13)	0.0457 (5)	
C2	0.8256 (7)	0.0261 (4)	0.4078 (2)	0.0849 (12)	
H2	0.852141	−0.021362	0.367817	0.102*	
C3	0.7515 (3)	0.1328 (3)	0.39290 (15)	0.0527 (6)	
C4	0.7098 (6)	0.2002 (4)	0.4530 (2)	0.0784 (10)	
H4	0.657245	0.272468	0.446699	0.094*	
C5	0.7469 (7)	0.1593 (5)	0.5232 (2)	0.0969 (15)	
H5	0.719813	0.203753	0.564383	0.116*	
C6	0.8237 (7)	0.0529 (5)	0.5304 (3)	0.1011 (16)	
H6	0.850092	0.026908	0.577437	0.121*	
C2'	0.7089 (3)	0.1711 (2)	0.31480 (15)	0.0453 (5)	
H2'A	0.592381	0.146268	0.304605	0.054*	
C3'	0.8224 (4)	0.1185 (2)	0.25495 (17)	0.0606 (7)	
H3'A	0.827492	0.034133	0.261322	0.073*	
H3'B	0.772751	0.134164	0.207184	0.073*	
C4'	1.0008 (4)	0.1681 (3)	0.2570 (2)	0.0681 (8)	
H4'A	1.054458	0.147950	0.303297	0.082*	
H4'B	1.067557	0.134495	0.217513	0.082*	
C5'	0.9938 (4)	0.3002 (3)	0.2487 (2)	0.0693 (9)	
H5'A	1.107324	0.332288	0.251852	0.083*	
H5'B	0.947808	0.320006	0.200815	0.083*	
C6'	0.8839 (4)	0.3532 (2)	0.3083 (2)	0.0596 (7)	
H6'A	0.938763	0.342307	0.355476	0.072*	
H6'B	0.873433	0.436851	0.299725	0.072*	
C7	0.5649 (3)	0.3733 (2)	0.18179 (14)	0.0481 (5)	
C8	0.6399 (4)	0.4666 (3)	0.1460 (2)	0.0626 (7)	
H8	0.681949	0.529831	0.172646	0.075*	
C9	0.6520 (6)	0.4652 (4)	0.0700 (2)	0.0819 (11)	
H9	0.703039	0.527751	0.045869	0.098*	
C10	0.5884 (5)	0.3708 (4)	0.02895 (19)	0.0784 (10)	
C11	0.5151 (6)	0.2779 (4)	0.0668 (2)	0.0793 (11)	
H11	0.473604	0.213963	0.040632	0.095*	
C12	0.5027 (5)	0.2787 (3)	0.14224 (18)	0.0625 (7)	
H12	0.452790	0.215910	0.166656	0.075*	
C13	0.5936 (8)	0.3691 (6)	−0.0544 (2)	0.127 (2)	
H13A	0.667796	0.429881	−0.071637	0.190*	
H13B	0.481636	0.382083	−0.073425	0.190*	
H13C	0.634474	0.294420	−0.070964	0.190*	

### (S)-3-[1-(4-Methylphenyl)piperidin-2-yl]pyridine (2) Atomic displacement parameters (Å^2^)

**Table:**

	*U*^11^	*U*^22^	*U*^33^	*U*^12^	*U*^13^	*U*^23^
S1	0.0479 (3)	0.0526 (3)	0.0501 (3)	0.0144 (3)	−0.0047 (2)	−0.0035 (3)
O1	0.0437 (10)	0.1059 (19)	0.0667 (13)	0.0080 (11)	0.0036 (9)	0.0118 (12)
O2	0.0981 (18)	0.0566 (12)	0.0733 (13)	0.0353 (13)	−0.0174 (13)	−0.0188 (10)
N1	0.149 (4)	0.102 (3)	0.085 (3)	0.018 (3)	−0.024 (3)	0.037 (2)
N1'	0.0442 (10)	0.0379 (10)	0.0550 (11)	0.0017 (8)	−0.0103 (9)	−0.0008 (9)
C2	0.117 (3)	0.067 (2)	0.070 (2)	0.016 (2)	−0.006 (2)	0.0198 (18)
C3	0.0557 (14)	0.0492 (14)	0.0533 (13)	−0.0067 (11)	−0.0059 (11)	0.0056 (12)
C4	0.099 (3)	0.080 (2)	0.0566 (18)	0.004 (2)	−0.0004 (18)	0.0021 (17)
C5	0.120 (4)	0.117 (4)	0.0541 (19)	−0.014 (3)	0.002 (2)	−0.003 (2)
C6	0.115 (4)	0.118 (4)	0.070 (3)	−0.024 (3)	−0.027 (2)	0.036 (3)
C2'	0.0486 (12)	0.0361 (11)	0.0512 (13)	−0.0043 (9)	−0.0080 (10)	0.0012 (10)
C3'	0.0848 (19)	0.0404 (12)	0.0565 (14)	0.0077 (13)	−0.0022 (14)	−0.0022 (12)
C4'	0.0637 (16)	0.0616 (16)	0.079 (2)	0.0210 (14)	0.0109 (15)	0.0129 (15)
C5'	0.0460 (13)	0.0626 (17)	0.099 (2)	0.0015 (12)	0.0014 (14)	0.0232 (17)
C6'	0.0530 (14)	0.0388 (12)	0.087 (2)	−0.0075 (11)	−0.0178 (14)	0.0043 (12)
C7	0.0443 (12)	0.0464 (12)	0.0536 (13)	0.0084 (11)	−0.0065 (10)	0.0012 (11)
C8	0.0639 (17)	0.0525 (15)	0.0714 (18)	−0.0007 (13)	−0.0036 (15)	0.0082 (14)
C9	0.082 (2)	0.087 (3)	0.077 (2)	0.011 (2)	0.0118 (19)	0.030 (2)
C10	0.087 (2)	0.094 (3)	0.0540 (16)	0.029 (2)	0.0010 (15)	0.0079 (18)
C11	0.101 (3)	0.075 (2)	0.0610 (18)	0.013 (2)	−0.0191 (18)	−0.0132 (17)
C12	0.0765 (19)	0.0513 (14)	0.0596 (16)	−0.0014 (14)	−0.0153 (14)	0.0005 (13)
C13	0.146 (5)	0.179 (6)	0.056 (2)	0.054 (5)	0.010 (3)	0.012 (3)

### (S)-3-[1-(4-Methylphenyl)piperidin-2-yl]pyridine (2) Geometric parameters (Å, º)

**Table:**

S1—O1	1.431 (2)	C4'—C5'	1.516 (4)
S1—O2	1.436 (2)	C4'—H4'A	0.9700
S1—N1'	1.620 (2)	C4'—H4'B	0.9700
S1—C7	1.763 (3)	C5'—C6'	1.515 (5)
N1—C6	1.316 (7)	C5'—H5'A	0.9700
N1—C2	1.333 (5)	C5'—H5'B	0.9700
N1'—C6'	1.477 (3)	C6'—H6'A	0.9700
N1'—C2'	1.479 (3)	C6'—H6'B	0.9700
C2—C3	1.378 (5)	C7—C8	1.381 (4)
C2—H2	0.9300	C7—C12	1.387 (4)
C3—C4	1.376 (5)	C8—C9	1.386 (6)
C3—C2'	1.524 (4)	C8—H8	0.9300
C4—C5	1.390 (6)	C9—C10	1.404 (6)
C4—H4	0.9300	C9—H9	0.9300
C5—C6	1.364 (8)	C10—C11	1.391 (6)
C5—H5	0.9300	C10—C13	1.518 (5)
C6—H6	0.9300	C11—C12	1.376 (5)
C2'—C3'	1.533 (4)	C11—H11	0.9300
C2'—H2'A	0.9800	C12—H12	0.9300
C3'—C4'	1.518 (5)	C13—H13A	0.9600
C3'—H3'A	0.9700	C13—H13B	0.9600
C3'—H3'B	0.9700	C13—H13C	0.9600
			
O1—S1—O2	119.94 (17)	C5'—C4'—H4'B	109.8
O1—S1—N1'	106.52 (13)	C3'—C4'—H4'B	109.8
O2—S1—N1'	106.71 (13)	H4'A—C4'—H4'B	108.2
O1—S1—C7	107.73 (14)	C6'—C5'—C4'	110.3 (3)
O2—S1—C7	106.80 (15)	C6'—C5'—H5'A	109.6
N1'—S1—C7	108.78 (12)	C4'—C5'—H5'A	109.6
C6—N1—C2	116.4 (4)	C6'—C5'—H5'B	109.6
C6'—N1'—C2'	115.3 (2)	C4'—C5'—H5'B	109.6
C6'—N1'—S1	119.61 (18)	H5'A—C5'—H5'B	108.1
C2'—N1'—S1	120.58 (18)	N1'—C6'—C5'	112.5 (2)
N1—C2—C3	125.7 (4)	N1'—C6'—H6'A	109.1
N1—C2—H2	117.2	C5'—C6'—H6'A	109.1
C3—C2—H2	117.2	N1'—C6'—H6'B	109.1
C4—C3—C2	116.1 (3)	C5'—C6'—H6'B	109.1
C4—C3—C2'	121.8 (3)	H6'A—C6'—H6'B	107.8
C2—C3—C2'	122.0 (3)	C8—C7—C12	120.5 (3)
C3—C4—C5	119.5 (4)	C8—C7—S1	119.7 (2)
C3—C4—H4	120.3	C12—C7—S1	119.8 (2)
C5—C4—H4	120.3	C7—C8—C9	119.4 (3)
C6—C5—C4	118.7 (4)	C7—C8—H8	120.3
C6—C5—H5	120.6	C9—C8—H8	120.3
C4—C5—H5	120.6	C8—C9—C10	121.0 (4)
N1—C6—C5	123.7 (4)	C8—C9—H9	119.5
N1—C6—H6	118.2	C10—C9—H9	119.5
C5—C6—H6	118.2	C11—C10—C9	118.1 (3)
N1'—C2'—C3	109.1 (2)	C11—C10—C13	119.8 (4)
N1'—C2'—C3'	110.1 (2)	C9—C10—C13	122.2 (4)
C3—C2'—C3'	114.9 (2)	C12—C11—C10	121.2 (4)
N1'—C2'—H2'A	107.5	C12—C11—H11	119.4
C3—C2'—H2'A	107.5	C10—C11—H11	119.4
C3'—C2'—H2'A	107.5	C11—C12—C7	119.8 (3)
C4'—C3'—C2'	112.2 (2)	C11—C12—H12	120.1
C4'—C3'—H3'A	109.2	C7—C12—H12	120.1
C2'—C3'—H3'A	109.2	C10—C13—H13A	109.5
C4'—C3'—H3'B	109.2	C10—C13—H13B	109.5
C2'—C3'—H3'B	109.2	H13A—C13—H13B	109.5
H3'A—C3'—H3'B	107.9	C10—C13—H13C	109.5
C5'—C4'—C3'	109.5 (2)	H13A—C13—H13C	109.5
C5'—C4'—H4'A	109.8	H13B—C13—H13C	109.5
C3'—C4'—H4'A	109.8		
			
O1—S1—N1'—C6'	−171.2 (2)	C3—C2'—C3'—C4'	−70.5 (3)
O2—S1—N1'—C6'	−41.9 (3)	C2'—C3'—C4'—C5'	−57.7 (4)
C7—S1—N1'—C6'	73.0 (2)	C3'—C4'—C5'—C6'	57.1 (4)
O1—S1—N1'—C2'	33.7 (2)	C2'—N1'—C6'—C5'	51.9 (3)
O2—S1—N1'—C2'	162.9 (2)	S1—N1'—C6'—C5'	−104.5 (3)
C7—S1—N1'—C2'	−82.2 (2)	C4'—C5'—C6'—N1'	−54.2 (4)
C6—N1—C2—C3	0.2 (9)	O1—S1—C7—C8	150.1 (2)
N1—C2—C3—C4	−1.5 (7)	O2—S1—C7—C8	20.0 (3)
N1—C2—C3—C2'	−178.1 (4)	N1'—S1—C7—C8	−94.8 (2)
C2—C3—C4—C5	1.3 (6)	O1—S1—C7—C12	−31.5 (3)
C2'—C3—C4—C5	178.0 (4)	O2—S1—C7—C12	−161.6 (2)
C3—C4—C5—C6	−0.1 (7)	N1'—S1—C7—C12	83.6 (2)
C2—N1—C6—C5	1.2 (9)	C12—C7—C8—C9	0.3 (5)
C4—C5—C6—N1	−1.2 (9)	S1—C7—C8—C9	178.7 (3)
C6'—N1'—C2'—C3	76.8 (3)	C7—C8—C9—C10	0.4 (6)
S1—N1'—C2'—C3	−127.1 (2)	C8—C9—C10—C11	−0.9 (6)
C6'—N1'—C2'—C3'	−50.2 (3)	C8—C9—C10—C13	177.5 (4)
S1—N1'—C2'—C3'	106.0 (2)	C9—C10—C11—C12	0.9 (6)
C4—C3—C2'—N1'	31.3 (4)	C13—C10—C11—C12	−177.6 (4)
C2—C3—C2'—N1'	−152.3 (3)	C10—C11—C12—C7	−0.3 (6)
C4—C3—C2'—C3'	155.4 (3)	C8—C7—C12—C11	−0.3 (5)
C2—C3—C2'—C3'	−28.1 (4)	S1—C7—C12—C11	−178.7 (3)
N1'—C2'—C3'—C4'	53.1 (3)		

### (S)-3-[1-(4-Methylphenyl)piperidin-2-yl]pyridine (2) Hydrogen-bond geometry (Å, º)

**Table:**

*D*—H···*A*	*D*—H	H···*A*	*D*···*A*	*D*—H···*A*
C12—H12···O2^i^	0.93	2.62	3.474 (4)	152
C5′—H5′*A*···O1^ii^	0.97	2.51	3.369 (4)	147
C2′—H2′*A*···O1	0.98	2.37	2.884 (4)	112

Symmetry codes: (i) −*x*+1, *y*−1/2, −*z*+1/2; (ii) *x*+1, *y*, *z*.

### (S)-3-{1-[(4-Methoxyphenyl)sulfonyl]piperidin-2-yl}pyridine (3) Crystal data

**Table:**

C_17_H_20_N_2_O_3_S	*D*_x_ = 1.326 Mg m^−^^3^
*M**_r_* = 332.41	Cu *K*α radiation, λ = 1.54184 Å
Orthorhombic, *P*2_1_2_1_2_1_	Cell parameters from 3974 reflections
*a* = 7.9758 (16) Å	θ = 4.6–75.4°
*b* = 11.131 (2) Å	µ = 1.87 mm^−^^1^
*c* = 18.754 (4) Å	*T* = 297 K
*V* = 1664.9 (6) Å^3^	Prism, colorless
*Z* = 4	0.30 × 0.25 × 0.25 mm
*F*(000) = 704	

### (S)-3-{1-[(4-Methoxyphenyl)sulfonyl]piperidin-2-yl}pyridine (3) Data collection

**Table:**

Xcalibur, Ruby diffractometer	3427 independent reflections
Radiation source: Enhance (Cu) X-ray Source	2899 reflections with *I* > 2σ(*I*)
Detector resolution: 10.25 pixels mm^-1^	*R*_int_ = 0.048
ω scans	θ_max_ = 76.0°, θ_min_ = 4.6°
Absorption correction: multi-scan (SADABS; Krause *et al.*, 2015)	*h* = −9→9
*T*_min_ = 0.78, *T*_max_ = 1.00	*k* = −13→12
12142 measured reflections	*l* = −16→23

### (S)-3-{1-[(4-Methoxyphenyl)sulfonyl]piperidin-2-yl}pyridine (3) Refinement

**Table:**

Refinement on *F*^2^	Secondary atom site location: dual
Least-squares matrix: full	Hydrogen site location: inferred from neighbouring sites
*R*[*F*^2^ > 2σ(*F*^2^)] = 0.054	H-atom parameters constrained
*wR*(*F*^2^) = 0.133	*w* = 1/[σ^2^(*F*_o_^2^) + (0.0709*P*)^2^] where *P* = (*F*_o_^2^ + 2*F*_c_^2^)/3
*S* = 1.10	(Δ/σ)_max_ < 0.001
3427 reflections	Δρ_max_ = 0.23 e Å^−^^3^
209 parameters	Δρ_min_ = −0.53 e Å^−^^3^
0 restraints	Absolute structure: Flack *x* determined using 1030 quotients [(*I*^+^)-(*I*^-^)]/[(*I*^+^)+(*I*^-^)] (Parsons *et al.*, 2013)
Primary atom site location: dual	Absolute structure parameter: −0.014 (15)

### (S)-3-{1-[(4-Methoxyphenyl)sulfonyl]piperidin-2-yl}pyridine (3) Special details

**Table:**

Geometry. All esds (except the esd in the dihedral angle between two l.s. planes) are estimated using the full covariance matrix. The cell esds are taken into account individually in the estimation of esds in distances, angles and torsion angles; correlations between esds in cell parameters are only used when they are defined by crystal symmetry. An approximate (isotropic) treatment of cell esds is used for estimating esds involving l.s. planes.

### (S)-3-{1-[(4-Methoxyphenyl)sulfonyl]piperidin-2-yl}pyridine (3) Fractional atomic coordinates and isotropic or equivalent isotropic displacement parameters (Å^2^)

**Table:**

	*x*	*y*	*z*	*U*_iso_*/*U*_eq_	
S1	0.46400 (12)	0.61342 (10)	0.28240 (5)	0.0586 (3)	
O1	0.6090 (3)	0.6847 (4)	0.29802 (18)	0.0831 (10)	
O2	0.4573 (5)	0.4897 (3)	0.30408 (18)	0.0914 (12)	
O3	0.3669 (6)	0.6134 (4)	−0.02843 (18)	0.0992 (12)	
N1	0.1744 (9)	1.0089 (4)	0.4792 (3)	0.1096 (19)	
N1'	0.3076 (4)	0.6794 (3)	0.32116 (16)	0.0484 (7)	
C2	0.2009 (9)	0.9644 (5)	0.4140 (3)	0.0853 (16)	
H2	0.171996	1.012250	0.375206	0.102*	
C3	0.2682 (4)	0.8523 (3)	0.39991 (19)	0.0505 (8)	
C4	0.3119 (7)	0.7844 (5)	0.4577 (2)	0.0783 (13)	
H4	0.360841	0.709297	0.451587	0.094*	
C5	0.2817 (9)	0.8299 (6)	0.5265 (3)	0.0959 (19)	
H5	0.308423	0.784710	0.566621	0.115*	
C6	0.2137 (9)	0.9398 (6)	0.5333 (3)	0.098 (2)	
H6	0.193347	0.968466	0.579069	0.118*	
C2'	0.2998 (4)	0.8114 (3)	0.32333 (19)	0.0474 (8)	
H2'A	0.409873	0.842128	0.308922	0.057*	
C3'	0.1712 (6)	0.8573 (4)	0.2696 (2)	0.0635 (10)	
H3'A	0.160356	0.943672	0.274506	0.076*	
H3'B	0.210472	0.840398	0.221716	0.076*	
C4'	0.0021 (5)	0.7995 (5)	0.2804 (3)	0.0758 (13)	
H4'A	−0.075740	0.828800	0.244638	0.091*	
H4'B	−0.041450	0.820735	0.327007	0.091*	
C5'	0.0179 (5)	0.6647 (5)	0.2745 (3)	0.0803 (14)	
H5'A	−0.090207	0.627923	0.283817	0.096*	
H5'B	0.051468	0.643487	0.226458	0.096*	
C6'	0.1448 (5)	0.6170 (4)	0.3267 (3)	0.0680 (11)	
H6'A	0.101579	0.626174	0.374716	0.082*	
H6'B	0.161359	0.531918	0.318011	0.082*	
C7	0.4342 (4)	0.6160 (4)	0.1891 (2)	0.0527 (8)	
C8	0.3478 (6)	0.5245 (4)	0.1551 (2)	0.0629 (10)	
H8	0.303865	0.461422	0.181689	0.076*	
C9	0.3268 (7)	0.5258 (4)	0.0837 (3)	0.0723 (12)	
H9	0.269326	0.463568	0.061386	0.087*	
C10	0.3910 (6)	0.6203 (4)	0.0433 (2)	0.0707 (11)	
C11	0.4770 (7)	0.7131 (4)	0.0767 (2)	0.0754 (13)	
H11	0.520194	0.776552	0.050174	0.091*	
C12	0.4977 (6)	0.7099 (4)	0.1497 (2)	0.0645 (11)	
H12	0.554942	0.771753	0.172491	0.077*	
C13	0.4504 (13)	0.6978 (6)	−0.0724 (3)	0.126 (3)	
H13A	0.428234	0.679622	−0.121564	0.189*	
H13B	0.568898	0.693693	−0.063780	0.189*	
H13C	0.410630	0.777137	−0.061717	0.189*	

### (S)-3-{1-[(4-Methoxyphenyl)sulfonyl]piperidin-2-yl}pyridine (3) Atomic displacement parameters (Å^2^)

**Table:**

	*U*^11^	*U*^22^	*U*^33^	*U*^12^	*U*^13^	*U*^23^
S1	0.0518 (4)	0.0659 (6)	0.0579 (4)	0.0238 (4)	0.0040 (4)	0.0017 (5)
O1	0.0406 (14)	0.132 (3)	0.076 (2)	0.0124 (16)	−0.0029 (13)	−0.0161 (19)
O2	0.127 (3)	0.068 (2)	0.078 (2)	0.055 (2)	0.016 (2)	0.0145 (16)
O3	0.139 (4)	0.100 (3)	0.0585 (17)	0.005 (3)	−0.005 (2)	−0.005 (2)
N1	0.171 (6)	0.083 (3)	0.074 (3)	0.009 (4)	0.028 (3)	−0.023 (3)
N1'	0.0449 (15)	0.0412 (15)	0.0591 (17)	0.0035 (12)	0.0085 (13)	0.0000 (13)
C2	0.130 (5)	0.060 (3)	0.065 (3)	0.014 (3)	0.015 (3)	−0.010 (2)
C3	0.0511 (18)	0.049 (2)	0.0517 (17)	−0.0082 (15)	0.0032 (14)	−0.0040 (15)
C4	0.095 (3)	0.079 (3)	0.061 (2)	0.008 (3)	−0.012 (2)	−0.003 (2)
C5	0.126 (5)	0.107 (5)	0.055 (2)	−0.014 (4)	−0.014 (3)	0.002 (3)
C6	0.125 (5)	0.099 (4)	0.069 (3)	−0.037 (4)	0.024 (3)	−0.027 (3)
C2'	0.0460 (17)	0.0416 (18)	0.0546 (19)	−0.0007 (14)	0.0090 (14)	−0.0014 (15)
C3'	0.086 (3)	0.052 (2)	0.0524 (19)	0.0177 (19)	0.0015 (18)	0.0036 (16)
C4'	0.058 (2)	0.099 (3)	0.070 (2)	0.030 (2)	−0.011 (2)	−0.022 (3)
C5'	0.0406 (18)	0.092 (3)	0.108 (4)	−0.0055 (19)	0.004 (2)	−0.041 (3)
C6'	0.065 (2)	0.0450 (19)	0.094 (3)	−0.0125 (19)	0.028 (2)	−0.007 (2)
C7	0.052 (2)	0.0500 (19)	0.0566 (17)	0.0104 (16)	0.0106 (14)	−0.0024 (17)
C8	0.062 (2)	0.053 (2)	0.074 (3)	−0.0044 (18)	0.0116 (19)	−0.0008 (19)
C9	0.078 (3)	0.065 (3)	0.074 (3)	−0.010 (2)	0.000 (2)	−0.011 (2)
C10	0.086 (3)	0.068 (3)	0.058 (2)	0.013 (3)	0.004 (2)	−0.007 (2)
C11	0.105 (4)	0.056 (2)	0.066 (2)	−0.003 (2)	0.021 (3)	0.004 (2)
C12	0.081 (3)	0.048 (2)	0.064 (2)	−0.007 (2)	0.013 (2)	−0.0084 (18)
C13	0.215 (9)	0.103 (4)	0.061 (3)	0.023 (5)	0.019 (4)	0.009 (3)

### (S)-3-{1-[(4-Methoxyphenyl)sulfonyl]piperidin-2-yl}pyridine (3) Geometric parameters (Å, º)

**Table:**

S1—O1	1.433 (4)	C3'—H3'B	0.9700
S1—O2	1.437 (4)	C4'—C5'	1.510 (7)
S1—N1'	1.620 (3)	C4'—H4'A	0.9700
S1—C7	1.765 (4)	C4'—H4'B	0.9700
O3—C10	1.360 (5)	C5'—C6'	1.504 (7)
O3—C13	1.416 (8)	C5'—H5'A	0.9700
N1—C6	1.311 (8)	C5'—H5'B	0.9700
N1—C2	1.337 (6)	C6'—H6'A	0.9700
N1'—C2'	1.471 (4)	C6'—H6'B	0.9700
N1'—C6'	1.477 (5)	C7—C12	1.377 (6)
C2—C3	1.384 (6)	C7—C8	1.386 (6)
C2—H2	0.9300	C8—C9	1.349 (7)
C3—C4	1.365 (6)	C8—H8	0.9300
C3—C2'	1.528 (5)	C9—C10	1.394 (7)
C4—C5	1.408 (7)	C9—H9	0.9300
C4—H4	0.9300	C10—C11	1.389 (7)
C5—C6	1.344 (9)	C11—C12	1.380 (6)
C5—H5	0.9300	C11—H11	0.9300
C6—H6	0.9300	C12—H12	0.9300
C2'—C3'	1.525 (5)	C13—H13A	0.9600
C2'—H2'A	0.9800	C13—H13B	0.9600
C3'—C4'	1.508 (7)	C13—H13C	0.9600
C3'—H3'A	0.9700		
			
O1—S1—O2	120.2 (2)	C5'—C4'—H4'A	109.7
O1—S1—N1'	106.19 (18)	C3'—C4'—H4'B	109.7
O2—S1—N1'	106.21 (19)	C5'—C4'—H4'B	109.7
O1—S1—C7	107.59 (19)	H4'A—C4'—H4'B	108.2
O2—S1—C7	106.9 (2)	C6'—C5'—C4'	111.1 (4)
N1'—S1—C7	109.47 (17)	C6'—C5'—H5'A	109.4
C10—O3—C13	118.1 (5)	C4'—C5'—H5'A	109.4
C6—N1—C2	116.9 (5)	C6'—C5'—H5'B	109.4
C2'—N1'—C6'	115.5 (3)	C4'—C5'—H5'B	109.4
C2'—N1'—S1	119.9 (2)	H5'A—C5'—H5'B	108.0
C6'—N1'—S1	119.7 (3)	N1'—C6'—C5'	112.3 (4)
N1—C2—C3	124.7 (5)	N1'—C6'—H6'A	109.1
N1—C2—H2	117.6	C5'—C6'—H6'A	109.1
C3—C2—H2	117.6	N1'—C6'—H6'B	109.1
C4—C3—C2	116.5 (4)	C5'—C6'—H6'B	109.1
C4—C3—C2'	122.6 (4)	H6'A—C6'—H6'B	107.9
C2—C3—C2'	120.8 (4)	C12—C7—C8	119.5 (4)
C3—C4—C5	119.0 (5)	C12—C7—S1	119.7 (3)
C3—C4—H4	120.5	C8—C7—S1	120.8 (3)
C5—C4—H4	120.5	C9—C8—C7	120.7 (4)
C6—C5—C4	118.9 (6)	C9—C8—H8	119.6
C6—C5—H5	120.5	C7—C8—H8	119.6
C4—C5—H5	120.5	C8—C9—C10	120.1 (4)
N1—C6—C5	123.8 (5)	C8—C9—H9	119.9
N1—C6—H6	118.1	C10—C9—H9	119.9
C5—C6—H6	118.1	O3—C10—C11	123.9 (5)
N1'—C2'—C3'	110.1 (3)	O3—C10—C9	116.3 (5)
N1'—C2'—C3	109.3 (3)	C11—C10—C9	119.8 (4)
C3'—C2'—C3	114.2 (3)	C12—C11—C10	119.2 (4)
N1'—C2'—H2'A	107.7	C12—C11—H11	120.4
C3'—C2'—H2'A	107.7	C10—C11—H11	120.4
C3—C2'—H2'A	107.7	C7—C12—C11	120.6 (4)
C4'—C3'—C2'	111.8 (3)	C7—C12—H12	119.7
C4'—C3'—H3'A	109.3	C11—C12—H12	119.7
C2'—C3'—H3'A	109.3	O3—C13—H13A	109.5
C4'—C3'—H3'B	109.3	O3—C13—H13B	109.5
C2'—C3'—H3'B	109.3	H13A—C13—H13B	109.5
H3'A—C3'—H3'B	107.9	O3—C13—H13C	109.5
C3'—C4'—C5'	109.8 (3)	H13A—C13—H13C	109.5
C3'—C4'—H4'A	109.7	H13B—C13—H13C	109.5
			
O1—S1—N1'—C2'	37.2 (4)	C2'—C3'—C4'—C5'	−57.7 (5)
O2—S1—N1'—C2'	166.2 (3)	C3'—C4'—C5'—C6'	56.3 (5)
C7—S1—N1'—C2'	−78.7 (3)	C2'—N1'—C6'—C5'	50.9 (5)
O1—S1—N1'—C6'	−168.7 (3)	S1—N1'—C6'—C5'	−104.3 (4)
O2—S1—N1'—C6'	−39.7 (4)	C4'—C5'—C6'—N1'	−52.4 (5)
C7—S1—N1'—C6'	75.4 (3)	O1—S1—C7—C12	−24.0 (4)
C6—N1—C2—C3	−0.9 (11)	O2—S1—C7—C12	−154.3 (3)
N1—C2—C3—C4	−0.9 (9)	N1'—S1—C7—C12	91.0 (3)
N1—C2—C3—C2'	−177.6 (6)	O1—S1—C7—C8	155.9 (3)
C2—C3—C4—C5	1.9 (8)	O2—S1—C7—C8	25.6 (4)
C2'—C3—C4—C5	178.6 (5)	N1'—S1—C7—C8	−89.1 (3)
C3—C4—C5—C6	−1.2 (9)	C12—C7—C8—C9	0.7 (6)
C2—N1—C6—C5	1.7 (11)	S1—C7—C8—C9	−179.2 (4)
C4—C5—C6—N1	−0.6 (11)	C7—C8—C9—C10	−0.5 (8)
C6'—N1'—C2'—C3'	−50.7 (4)	C13—O3—C10—C11	7.6 (8)
S1—N1'—C2'—C3'	104.4 (3)	C13—O3—C10—C9	−171.0 (6)
C6'—N1'—C2'—C3	75.5 (4)	C8—C9—C10—O3	178.8 (5)
S1—N1'—C2'—C3	−129.4 (3)	C8—C9—C10—C11	0.1 (8)
C4—C3—C2'—N1'	25.2 (5)	O3—C10—C11—C12	−178.5 (5)
C2—C3—C2'—N1'	−158.3 (4)	C9—C10—C11—C12	0.1 (8)
C4—C3—C2'—C3'	149.1 (4)	C8—C7—C12—C11	−0.5 (6)
C2—C3—C2'—C3'	−34.4 (6)	S1—C7—C12—C11	179.4 (4)
N1'—C2'—C3'—C4'	54.0 (4)	C10—C11—C12—C7	0.1 (7)
C3—C2'—C3'—C4'	−69.4 (4)		

### (S)-3-{1-[(4-Methoxyphenyl)sulfonyl]piperidin-2-yl}pyridine (3) Hydrogen-bond geometry (Å, º)

**Table:**

*D*—H···*A*	*D*—H	H···*A*	*D*···*A*	*D*—H···*A*
C12—H12···O2^i^	0.93	2.47	3.252 (5)	142
C5′—H5′*A*···O1^ii^	0.97	2.49	3.298 (5)	140
C2′—H2′*A*···O1	0.98	2.37	2.880 (5)	111

Symmetry codes: (i) −*x*+1, *y*+1/2, −*z*+1/2; (ii) *x*−1, *y*, *z*.

### (S)-3-{1-[(3,4-Dimethylphenyl)sulfonyl]piperidin-2-yl}pyridine (4) Crystal data

**Table:**

C_18_H_22_N_2_O_2_S	*D*_x_ = 1.305 Mg m^−^^3^
*M**_r_* = 330.43	Cu *K*α radiation, λ = 1.54184 Å
Orthorhombic, *P*2_1_2_1_2_1_	Cell parameters from 3960 reflections
*a* = 7.9087 (16) Å	θ = 3.8–75.1°
*b* = 11.737 (2) Å	µ = 1.80 mm^−^^1^
*c* = 18.117 (4) Å	*T* = 295 K
*V* = 1681.6 (6) Å^3^	Prism, colourless
*Z* = 4	0.45 × 0.25 × 0.22 mm
*F*(000) = 704	

### (S)-3-{1-[(3,4-Dimethylphenyl)sulfonyl]piperidin-2-yl}pyridine (4) Data collection

**Table:**

Xcalibur, Ruby diffractometer	3428 independent reflections
Radiation source: Enhance (Cu) X-ray Source	2886 reflections with *I* > 2σ(*I*)
Detector resolution: 10.25 pixels mm^-1^	*R*_int_ = 0.045
ω scans	θ_max_ = 76.2°, θ_min_ = 4.5°
Absorption correction: multi-scan (SADABS; Krause *et al.*, 2015)	*h* = −9→9
*T*_min_ = 0.88, *T*_max_ = 1.00	*k* = −14→14
12220 measured reflections	*l* = −22→16

### (S)-3-{1-[(3,4-Dimethylphenyl)sulfonyl]piperidin-2-yl}pyridine (4) Refinement

**Table:**

Refinement on *F*^2^	Secondary atom site location: dual
Least-squares matrix: full	Hydrogen site location: inferred from neighbouring sites
*R*[*F*^2^ > 2σ(*F*^2^)] = 0.045	H-atom parameters constrained
*wR*(*F*^2^) = 0.111	*w* = 1/[σ^2^(*F*_o_^2^) + (0.0581*P*)^2^] where *P* = (*F*_o_^2^ + 2*F*_c_^2^)/3
*S* = 1.07	(Δ/σ)_max_ < 0.001
3428 reflections	Δρ_max_ = 0.21 e Å^−^^3^
210 parameters	Δρ_min_ = −0.34 e Å^−^^3^
0 restraints	Absolute structure: Flack *x* determined using 1007 quotients [(*I*^+^)-(*I*^-^)]/[(*I*^+^)+(*I*^-^)] (Parsons *et al.*, 2013)
Primary atom site location: dual	Absolute structure parameter: 0.011 (14)

### (S)-3-{1-[(3,4-Dimethylphenyl)sulfonyl]piperidin-2-yl}pyridine (4) Special details

**Table:**

Geometry. All esds (except the esd in the dihedral angle between two l.s. planes) are estimated using the full covariance matrix. The cell esds are taken into account individually in the estimation of esds in distances, angles and torsion angles; correlations between esds in cell parameters are only used when they are defined by crystal symmetry. An approximate (isotropic) treatment of cell esds is used for estimating esds involving l.s. planes.

### (S)-3-{1-[(3,4-Dimethylphenyl)sulfonyl]piperidin-2-yl}pyridine (4) Fractional atomic coordinates and isotropic or equivalent isotropic displacement parameters (Å^2^)

**Table:**

	*x*	*y*	*z*	*U*_iso_*/*U*_eq_	
S1	0.44287 (11)	0.14787 (7)	0.28526 (4)	0.0513 (2)	
O1	0.5899 (3)	0.2085 (3)	0.30954 (14)	0.0713 (8)	
O2	0.4152 (4)	0.0330 (2)	0.30906 (14)	0.0738 (8)	
N1	0.1338 (6)	0.5418 (3)	0.4672 (2)	0.0802 (11)	
N1'	0.2807 (3)	0.2215 (2)	0.31248 (14)	0.0459 (6)	
C2	0.1745 (6)	0.4979 (3)	0.4014 (2)	0.0648 (10)	
H2	0.157045	0.543351	0.360070	0.078*	
C3	0.2404 (4)	0.3903 (3)	0.39001 (17)	0.0471 (7)	
C4	0.2700 (6)	0.3259 (3)	0.45187 (19)	0.0660 (10)	
H4	0.318057	0.253834	0.447733	0.079*	
C5	0.2273 (6)	0.3694 (4)	0.5209 (2)	0.0775 (12)	
H5	0.244599	0.326389	0.563353	0.093*	
C6	0.1599 (6)	0.4756 (4)	0.5252 (2)	0.0762 (13)	
H6	0.130429	0.503421	0.571493	0.091*	
C2'	0.2876 (4)	0.3471 (3)	0.31322 (16)	0.0458 (7)	
H2'A	0.404956	0.369480	0.303765	0.055*	
C3'	0.1791 (6)	0.3954 (3)	0.25051 (18)	0.0601 (9)	
H3'A	0.175535	0.477799	0.254520	0.072*	
H3'B	0.230610	0.376342	0.203524	0.072*	
C4'	0.0008 (5)	0.3491 (4)	0.2525 (2)	0.0687 (10)	
H4'B	−0.054026	0.372414	0.297957	0.082*	
H4'A	−0.063361	0.379805	0.211468	0.082*	
C5'	0.0037 (5)	0.2213 (4)	0.2479 (2)	0.0685 (11)	
H5'B	0.050165	0.198218	0.200642	0.082*	
H5'A	−0.110794	0.192194	0.251255	0.082*	
C6'	0.1102 (5)	0.1715 (3)	0.3099 (2)	0.0585 (9)	
H6'A	0.053836	0.184907	0.356662	0.070*	
H6'B	0.119525	0.089753	0.303072	0.070*	
C7	0.4458 (4)	0.1468 (3)	0.18773 (16)	0.0478 (6)	
C8	0.3742 (5)	0.0569 (3)	0.1500 (2)	0.0536 (8)	
H8	0.326167	−0.003003	0.176245	0.064*	
C9	0.3728 (5)	0.0547 (4)	0.0731 (2)	0.0662 (11)	
C10	0.4447 (6)	0.1471 (4)	0.03484 (19)	0.0725 (11)	
C11	0.5126 (6)	0.2369 (4)	0.0740 (2)	0.0727 (12)	
H11	0.558208	0.298156	0.048268	0.087*	
C12	0.5152 (5)	0.2386 (3)	0.1501 (2)	0.0609 (9)	
H12	0.562128	0.299646	0.175608	0.073*	
C13	0.4524 (9)	0.1493 (6)	−0.0490 (2)	0.117 (2)	
H13A	0.465980	0.226429	−0.065598	0.176*	
H13B	0.349504	0.118467	−0.068832	0.176*	
H13C	0.546474	0.104368	−0.065538	0.176*	
C14	0.2982 (7)	−0.0467 (5)	0.0344 (3)	0.1006 (17)	
H14A	0.242833	−0.094837	0.069736	0.151*	
H14B	0.386563	−0.088725	0.010278	0.151*	
H14C	0.217586	−0.021386	−0.001693	0.151*	

### (S)-3-{1-[(3,4-Dimethylphenyl)sulfonyl]piperidin-2-yl}pyridine (4) Atomic displacement parameters (Å^2^)

**Table:**

	*U*^11^	*U*^22^	*U*^33^	*U*^12^	*U*^13^	*U*^23^
S1	0.0538 (4)	0.0543 (4)	0.0457 (3)	0.0128 (4)	−0.0003 (4)	0.0025 (3)
O1	0.0490 (16)	0.103 (2)	0.0614 (14)	0.0111 (14)	−0.0086 (11)	−0.0105 (14)
O2	0.099 (2)	0.0579 (15)	0.0644 (14)	0.0281 (15)	0.0088 (15)	0.0157 (12)
N1	0.099 (3)	0.072 (2)	0.070 (2)	0.005 (2)	0.0139 (19)	−0.0233 (19)
N1'	0.0470 (15)	0.0423 (14)	0.0484 (13)	−0.0004 (12)	0.0055 (11)	−0.0010 (11)
C2	0.083 (3)	0.054 (2)	0.057 (2)	0.0003 (19)	0.0050 (19)	−0.0065 (17)
C3	0.0486 (18)	0.0468 (17)	0.0457 (16)	−0.0090 (13)	0.0018 (14)	−0.0031 (13)
C4	0.086 (3)	0.063 (2)	0.0494 (18)	0.003 (2)	0.0042 (18)	−0.0026 (16)
C5	0.096 (3)	0.091 (3)	0.0453 (18)	−0.005 (3)	0.0028 (19)	−0.005 (2)
C6	0.079 (3)	0.091 (3)	0.059 (2)	−0.014 (2)	0.012 (2)	−0.028 (2)
C2'	0.0528 (18)	0.0396 (15)	0.0449 (13)	−0.0040 (14)	0.0076 (13)	0.0001 (13)
C3'	0.085 (3)	0.0494 (19)	0.0461 (19)	0.0083 (17)	0.0047 (17)	0.0002 (14)
C4'	0.070 (2)	0.076 (3)	0.060 (2)	0.021 (2)	−0.0114 (16)	−0.011 (2)
C5'	0.051 (2)	0.079 (3)	0.076 (2)	−0.0010 (18)	−0.0030 (17)	−0.024 (2)
C6'	0.057 (2)	0.0468 (18)	0.071 (2)	−0.0100 (14)	0.0121 (17)	−0.0071 (15)
C7	0.0470 (16)	0.0500 (16)	0.0463 (13)	0.0116 (17)	0.0035 (13)	−0.0027 (13)
C8	0.0516 (19)	0.0476 (18)	0.0615 (18)	0.0099 (14)	0.0050 (15)	−0.0060 (15)
C9	0.055 (2)	0.079 (3)	0.064 (2)	0.025 (2)	−0.0063 (18)	−0.021 (2)
C10	0.076 (3)	0.094 (3)	0.0475 (16)	0.035 (3)	0.0031 (18)	0.000 (2)
C11	0.083 (3)	0.077 (3)	0.058 (2)	0.016 (2)	0.0150 (19)	0.017 (2)
C12	0.067 (3)	0.056 (2)	0.0602 (19)	0.0046 (17)	0.0099 (17)	0.0030 (17)
C13	0.131 (5)	0.174 (6)	0.046 (2)	0.070 (5)	0.002 (3)	0.002 (3)
C14	0.086 (4)	0.115 (4)	0.100 (3)	0.016 (3)	−0.012 (3)	−0.056 (3)

### (S)-3-{1-[(3,4-Dimethylphenyl)sulfonyl]piperidin-2-yl}pyridine (4) Geometric parameters (Å, º)

**Table:**

S1—O1	1.432 (3)	C4'—H4'A	0.9700
S1—O2	1.432 (3)	C5'—C6'	1.521 (6)
S1—N1'	1.623 (3)	C5'—H5'B	0.9700
S1—C7	1.767 (3)	C5'—H5'A	0.9700
N1—C6	1.322 (6)	C6'—H6'A	0.9700
N1—C2	1.339 (5)	C6'—H6'B	0.9700
N1'—C6'	1.472 (4)	C7—C8	1.378 (5)
N1'—C2'	1.475 (4)	C7—C12	1.387 (5)
C2—C3	1.381 (5)	C8—C9	1.394 (5)
C2—H2	0.9300	C8—H8	0.9300
C3—C4	1.372 (5)	C9—C10	1.407 (6)
C3—C2'	1.527 (4)	C9—C14	1.503 (6)
C4—C5	1.392 (5)	C10—C11	1.379 (7)
C4—H4	0.9300	C10—C13	1.520 (5)
C5—C6	1.359 (7)	C11—C12	1.380 (5)
C5—H5	0.9300	C11—H11	0.9300
C6—H6	0.9300	C12—H12	0.9300
C2'—C3'	1.532 (5)	C13—H13A	0.9600
C2'—H2'A	0.9800	C13—H13B	0.9600
C3'—C4'	1.511 (6)	C13—H13C	0.9600
C3'—H3'A	0.9700	C14—H14A	0.9600
C3'—H3'B	0.9700	C14—H14B	0.9600
C4'—C5'	1.502 (6)	C14—H14C	0.9600
C4'—H4'B	0.9700		
			
O1—S1—O2	119.96 (19)	C4'—C5'—C6'	110.6 (3)
O1—S1—N1'	106.46 (15)	C4'—C5'—H5'B	109.5
O2—S1—N1'	106.79 (17)	C6'—C5'—H5'B	109.5
O1—S1—C7	107.46 (17)	C4'—C5'—H5'A	109.5
O2—S1—C7	107.23 (16)	C6'—C5'—H5'A	109.5
N1'—S1—C7	108.54 (14)	H5'B—C5'—H5'A	108.1
C6—N1—C2	116.3 (4)	N1'—C6'—C5'	112.2 (3)
C6'—N1'—C2'	115.6 (3)	N1'—C6'—H6'A	109.2
C6'—N1'—S1	120.2 (2)	C5'—C6'—H6'A	109.2
C2'—N1'—S1	120.4 (2)	N1'—C6'—H6'B	109.2
N1—C2—C3	125.2 (4)	C5'—C6'—H6'B	109.2
N1—C2—H2	117.4	H6'A—C6'—H6'B	107.9
C3—C2—H2	117.4	C8—C7—C12	120.9 (3)
C4—C3—C2	116.5 (3)	C8—C7—S1	119.7 (3)
C4—C3—C2'	121.3 (3)	C12—C7—S1	119.4 (3)
C2—C3—C2'	122.2 (3)	C7—C8—C9	120.9 (4)
C3—C4—C5	119.3 (4)	C7—C8—H8	119.6
C3—C4—H4	120.3	C9—C8—H8	119.6
C5—C4—H4	120.3	C8—C9—C10	118.4 (4)
C6—C5—C4	118.9 (4)	C8—C9—C14	119.0 (5)
C6—C5—H5	120.6	C10—C9—C14	122.6 (4)
C4—C5—H5	120.6	C11—C10—C9	119.5 (3)
N1—C6—C5	123.7 (4)	C11—C10—C13	119.0 (5)
N1—C6—H6	118.1	C9—C10—C13	121.4 (5)
C5—C6—H6	118.1	C10—C11—C12	122.1 (4)
N1'—C2'—C3	109.4 (2)	C10—C11—H11	119.0
N1'—C2'—C3'	110.0 (3)	C12—C11—H11	119.0
C3—C2'—C3'	114.5 (3)	C11—C12—C7	118.3 (4)
N1'—C2'—H2'A	107.5	C11—C12—H12	120.9
C3—C2'—H2'A	107.5	C7—C12—H12	120.9
C3'—C2'—H2'A	107.5	C10—C13—H13A	109.5
C4'—C3'—C2'	111.8 (3)	C10—C13—H13B	109.5
C4'—C3'—H3'A	109.3	H13A—C13—H13B	109.5
C2'—C3'—H3'A	109.3	C10—C13—H13C	109.5
C4'—C3'—H3'B	109.3	H13A—C13—H13C	109.5
C2'—C3'—H3'B	109.3	H13B—C13—H13C	109.5
H3'A—C3'—H3'B	107.9	C9—C14—H14A	109.5
C5'—C4'—C3'	110.1 (3)	C9—C14—H14B	109.5
C5'—C4'—H4'B	109.6	H14A—C14—H14B	109.5
C3'—C4'—H4'B	109.6	C9—C14—H14C	109.5
C5'—C4'—H4'A	109.6	H14A—C14—H14C	109.5
C3'—C4'—H4'A	109.6	H14B—C14—H14C	109.5
H4'B—C4'—H4'A	108.2		
			
O1—S1—N1'—C6'	−167.4 (3)	C2'—C3'—C4'—C5'	−57.8 (4)
O2—S1—N1'—C6'	−38.2 (3)	C3'—C4'—C5'—C6'	56.9 (4)
C7—S1—N1'—C6'	77.2 (3)	C2'—N1'—C6'—C5'	51.4 (4)
O1—S1—N1'—C2'	35.6 (3)	S1—N1'—C6'—C5'	−106.6 (3)
O2—S1—N1'—C2'	164.9 (2)	C4'—C5'—C6'—N1'	−53.3 (4)
C7—S1—N1'—C2'	−79.8 (3)	O1—S1—C7—C8	150.0 (3)
C6—N1—C2—C3	0.1 (7)	O2—S1—C7—C8	19.8 (3)
N1—C2—C3—C4	−1.9 (7)	N1'—S1—C7—C8	−95.3 (3)
N1—C2—C3—C2'	−178.6 (4)	O1—S1—C7—C12	−32.2 (3)
C2—C3—C4—C5	2.3 (6)	O2—S1—C7—C12	−162.5 (3)
C2'—C3—C4—C5	179.0 (4)	N1'—S1—C7—C12	82.5 (3)
C3—C4—C5—C6	−1.1 (7)	C12—C7—C8—C9	1.4 (5)
C2—N1—C6—C5	1.4 (7)	S1—C7—C8—C9	179.1 (3)
C4—C5—C6—N1	−0.9 (8)	C7—C8—C9—C10	−0.7 (5)
C6'—N1'—C2'—C3	76.0 (4)	C7—C8—C9—C14	178.1 (4)
S1—N1'—C2'—C3	−126.1 (2)	C8—C9—C10—C11	−0.5 (6)
C6'—N1'—C2'—C3'	−50.6 (4)	C14—C9—C10—C11	−179.3 (4)
S1—N1'—C2'—C3'	107.3 (3)	C8—C9—C10—C13	178.3 (4)
C4—C3—C2'—N1'	28.4 (5)	C14—C9—C10—C13	−0.5 (7)
C2—C3—C2'—N1'	−155.1 (3)	C9—C10—C11—C12	1.0 (7)
C4—C3—C2'—C3'	152.5 (4)	C13—C10—C11—C12	−177.7 (4)
C2—C3—C2'—C3'	−31.0 (5)	C10—C11—C12—C7	−0.4 (6)
N1'—C2'—C3'—C4'	53.3 (4)	C8—C7—C12—C11	−0.9 (6)
C3—C2'—C3'—C4'	−70.3 (4)	S1—C7—C12—C11	−178.6 (3)

### (S)-3-{1-[(3,4-Dimethylphenyl)sulfonyl]piperidin-2-yl}pyridine (4) Hydrogen-bond geometry (Å, º)

**Table:**

*D*—H···*A*	*D*—H	H···*A*	*D*···*A*	*D*—H···*A*
C5′—H5′*A*···O1^i^	0.97	2.60	3.462 (5)	148
C5—H5···O1^ii^	0.93	2.64	3.384 (5)	138
C2′—H2′*A*···O1	0.98	2.39	2.892 (4)	111

Symmetry codes: (i) *x*−1, *y*, *z*; (ii) *x*−1/2, −*y*+1/2, −*z*+1.
